# Forecasting alcohol lapse risk up to two weeks in advance using time-lagged machine learning models

**DOI:** 10.1371/journal.pone.0356893

**Published:** 2026-09-08

**Authors:** Kendra Wyant, Gaylen E. Fronk, Jiachen Yu, Claire E. Punturieri, John J. Curtin

**Affiliations:** 1 Department of Psychology, University of Wisconsin-Madison, Madison, Wisconsin, United States of America; 2 Department of Psychiatry and Behavioral Sciences, Medical University of South Carolina, Charleston, South Carolina, United States of America; University of Reading - Whiteknights Campus: University of Reading, UNITED KINGDOM OF GREAT BRITAIN AND NORTHERN IRELAND

## Abstract

Alcohol use disorder is a chronic, relapsing condition. Individuals must monitor risk and take proactive steps to manage and reduce it indefinitely to prevent lapse. Smartphone sensing and machine learning offer promise as scalable tools for automating risk monitoring and delivering personalized support during recovery. Research has demonstrated that machine learning models using ecological momentary assessment can generate highly accurate predictions of immediate lapse risk (e.g., within the next hour or day). However, many risks require supports that are not immediately available. These supports may need to be planned or involve coordination with others. In such cases, individuals may benefit from advance warning about changes in their lapse risk and the contributing factors. To meet this need, we developed machine learning models to predict future alcohol lapses within 24-hour prediction windows, lagged by 1 day, 3 days, 1 week, and 2 weeks from the prediction timepoint. We engineered features from 4x daily ecological momentary assessments from individuals (N = 151; 51% male; mean age = 41; 87% non-Hispanic White) in early recovery (≤ 8 weeks of abstinence) from alcohol use disorder over a three-month period. We trained and evaluated models using nested cross-validation. Median posterior auROC values were high (0.85–0.89) across models, though performance decreased modestly with longer lag time. Models performed worse for non-advantaged groups (non-White and/or Hispanic, income below federal poverty line, female) compared to advantaged groups (non-Hispanic White, income above federal poverty line, male). Past alcohol use, abstinence self-efficacy, and craving were the most important features, with the magnitude of their importance varying meaningfully by lag time. These findings demonstrate the feasibility of predicting alcohol lapses up to two weeks in advance. Embedding these models within a recovery monitoring and support system could enable adaptive, personalized care with enough warning to implement recovery supports not immediately available. Improving model fairness and optimizing the delivery of model feedback to sustain engagement remain critical next steps.

## Introduction

Alcohol and other substance use disorders (SUDs) are serious chronic conditions, characterized by high relapse rates [1,2], substantial co-morbidity with other physical and mental health problems [2,3], and an increased risk of mortality [4,5]. Too few individuals receive medications or clinician-delivered interventions to help them initially achieve abstinence and/or reduce harms associated with their use [3]. Moreover, this problem is even worse for subsequent continuing care during SUD recovery. Continuing care, including both risk monitoring and ongoing support, is the gold standard for managing chronic health conditions such as diabetes, asthma, and HIV [6]. Yet, continuing care for SUDs is largely lacking despite ample evidence that SUDs are chronic, relapsing conditions [3,7,8].

An important focus of continuing care during SUD recovery is to prevent lapses (i.e., single instances of goal-inconsistent substance use) and full relapse back to harmful use [9,10]. Critically, the risk factors that instigate lapses during recovery are individualized, numerous, dynamic, interactive, and non-linear [11,12]. The optimal supports to address these risk factors and encourage continued, successful recovery vary both across individuals and within an individual over time. Given this, continuing care could benefit greatly from a precision mental health approach that seeks to provide the right support to the right individual at the right time, every time [13–15]. However, such monitoring and personalized support must also be highly scalable to address the substantial unmet need for SUD continuing care.

Recent advances in both smartphone sensing [16] and machine learning [17] hold promise as a scalable foundation for monitoring and personalized support during SUD recovery. Smartphone sensing approaches (e.g., ecological momentary assessment [EMA], geolocation sensing) can provide the frequent, longitudinal measurement of proximal risk factors that is necessary for prediction of future lapses with high temporal precision. EMA may be particularly well-suited for lapse prediction because it can provide privileged access to subjective experiences (e.g., craving, affect, stress, motivation, self-efficacy) that are targets for change in evidence-based approaches for relapse prevention [9,10,18]. Furthermore, individuals with SUDs have found EMA to be acceptable for sustained measurement for up to a year with relatively high compliance [19,20], suggesting that this method is feasible for long-term monitoring throughout SUD recovery.

Machine learning models are well-positioned to use EMAs as inputs to provide temporally precise prediction of the probability of future lapses with sufficiently high performance to support decisions about interventions and other supports for specific individuals. These models can handle the high dimensional feature sets that may result from feature engineering densely sampled raw EMA over time [21]. They can also accommodate non-linear and interactive relationships between features and lapse probability that are likely necessary for accurate prediction of lapse probability. Moreover, rapid advances in the tools for interpretable machine learning (e.g., Shapley values [22]) now allow us to probe these models to understand which risk features contribute most strongly to a lapse prediction for a specific individual at a specific moment in time. Interventions, supports, and/or suggested lifestyle adjustments can then be personalized to address these risks following from our understanding about relapse prevention.

Preliminary research is now emerging that uses features derived from EMAs in machine learning models to predict the probability of future alcohol use [21,23,24]. This research is important because it rigorously required strict temporal ordering necessary for true prediction, with features measured before alcohol use outcomes. These studies also used resampling methods (e.g., cross-validation) that prioritize model generalizability to increase the likelihood these models will perform well with new people. Perhaps most importantly, Wyant et al. [21] demonstrated that machine learning models using EMA can provide predictions with very high temporal precision at clinically implementable levels of performance. Specifically, we developed models that predict lapses in the immediate future (i.e., the next day and even the next hour) with areas under the receiver operating characteristic curve (auROCs) of 0.91 and 0.93, respectively.

Wyant et al.’s [21] next day lapse prediction model can provide personalized support recommendations to address immediate risks for possible lapses. Features derived from past EMAs can be updated in the early morning to yield the predicted lapse probability for an individual that day. Personalized supports that target the top features contributing to that prediction can then be provided. For example, if predicted lapse probability is high due to frequent craving, the individual could be reminded about the benefits of urge surfing or distracting activities during brief periods when cravings arise. Conversely, guided relaxation techniques could be recommended if lapse probability is high due to recent past and anticipated stressors that day. Patients could also be assisted to implement any of these recommendations using videos or other tools within a digital therapeutic. Curtin and colleagues are currently evaluating outcomes associated with the use of this “smart” (machine learning guided) recovery monitoring and support system (RMSS) for patients with an alcohol use disorder (AUD) [25].

Despite the promise offered by a smart RMSS based on immediate future risks (e.g., the next day), such a system has limitations. Most importantly, recommendations must be limited to previously learned skills and/or supports that are available to implement that day. However, many risks may require supports that are not available in the moment. For example, to address lifestyle imbalances, several future positive activities may need to be planned. Time with supportive friends or an AA sponsor may require time to schedule. Similarly, work or family schedules may need to be adjusted to return to attending self-help meetings. If new recovery skills or therapeutic activities are needed to address emerging risks, patients may need to book sessions with a therapist. In all these instances, patients would benefit from advanced warning about changes in their lapse probability and the associated risks that contribute to these changes. A smart RMSS could provide this advanced warning by lagging lapse probability predictions further into the future (e.g., predicting lapse probability on a given day two weeks from now). However, we do not know if such lagged models could maintain adequate performance for clinical implementation.

In this study, we evaluated the performance of machine learning models that predict the probability of future lapses within 24-hour prediction windows that were systematically lagged further into the future. We considered several meaningful lags for these prediction windows: 1 day, 3 days, 1 week, and 2 weeks. We conducted pre-registered analyses of both the absolute performance of these lagged models and their relative performance compared to a baseline model that predicted lapse probability in the immediate next day (i.e., no lag). In addition to the aggregate performance of these models, we also evaluated algorithmic fairness by comparing model performance across important subgroups that have documented disparities in treatment access and/or outcomes. These include comparisons by race/ethnicity [26,27], income [28] and sex at birth [27,29]. Finally, we calculated Shapley values for feature categories defined by EMA items to better understand how these models generate predictions and how these features can be used to tailor personalized supports.

## Methods

### Transparency

We adhere to research transparency principles that are crucial for robust and replicable science. We preregistered our data analytic strategy. We reported all data exclusions. Our data, questionnaires, preregistration, analysis scripts, and other study materials are publicly available on our OSF page (https://osf.io/xta67/).

### Participants

We recruited 192 participants in early recovery (<= 8 weeks of abstinence) from AUD in Madison, Wisconsin, USA for a 3-month longitudinal study from February 15, 2017 through September 19, 2019. This sample size was determined based on traditional power analysis methods for logistic regression [30] because comparable approaches for machine learning models have not yet been validated. Participants were recruited through print and targeted digital advertisements and partnerships with treatment centers. We required that participants:

1 were age 18 or older,
2 could write and read in English,
3 had at least moderate AUD (>= 4 self-reported DSM-5 symptoms),
4 were abstinent from alcohol for 1–8 weeks, and
5 were willing to use a single smartphone (personal or study provided) while on study.

We also excluded participants exhibiting severe symptoms of psychosis or paranoia (defined as scores >2.2 or 2.8, respectively, on the psychosis or paranoia scales of the Symptom Checklist–90 [31]) because individuals experiencing severe psychotic symptoms may require a higher level of clinical support than the study protocol provided (e.g., with real-time clinical monitoring or treatment). We did not exclude participants based on the presence of severe symptoms of other mental health disorders because we aimed to recruit a sample representative of the broader AUD population, in which psychiatric disorders frequently co-occur.

Of the 192 eligible participants, 191 consented to participate in the study at the screening visit, and 169 subsequently enrolled in the study at the enrollment visit, which occurred approximately one week later. Fifteen participants discontinued before the first monthly follow-up visit. We excluded data from one participant who did not maintain a goal of abstinence during their participation. We also excluded data from two participants due to evidence of careless responding and unusually low compliance. Our final sample consisted of 151 participants.

### Ethics

All procedures were approved by the University of Wisconsin-Madison Institutional Review Board (Study #2015−0780) and carried out in accordance with the principles of the Declaration of Helsinki. All participants provided written informed consent observed by a research assistant.

### Procedure

Participants completed five study visits over approximately three months. After an initial phone screen, participants attended an in-person screening visit to determine eligibility, complete informed consent, and collect self-report measures. Eligible, consented participants returned approximately one week later for an intake visit. Three additional follow-up visits occurred about every 30 days that participants remained on study. Participants were expected to complete four daily EMAs. Other personal sensing data streams (geolocation, cellular communications, sleep quality, and audio check-ins) were collected as part of the parent grant’s aims (R01 AA024391).

Participants were compensated $20 per hour for all time spent in the laboratory (i.e., during screening, intake, and follow-up visits). In addition, participants received a $99 bonus upon completing the full 3-month study. They were also provided $66 per month to offset costs associated with their cellular plans. Participants received $25 for each month in which they provided EMA data with less than 10% missingness. Additional compensation was provided for other personal sensing data streams if minimum compliance thresholds were met.

### Measures Ecological momentary assessments

Participants completed four brief (7–10 questions) EMAs daily. The first and last EMAs of the day were scheduled within one hour of participants’ typical wake and sleep times. The other two EMAs were scheduled randomly within the first and second halves of their typical day, with at least one hour between EMAs. Participants learned how to complete the EMA and reviewed the meaning of each question with a member of the research team during their intake visit to ensure consistent question interpretation.

On the first item of all EMAs, participants reported the start and end dates and times of any unreported past alcohol use. Next, participants rated the maximum intensity of recent (i.e., since last EMA) experiences of craving, risky situations, stressful events, and pleasant events. Finally, participants rated their current affect on two bipolar scales: valence (Unpleasant/Unhappy to Pleasant/Happy) and arousal (Calm/Sleepy to Aroused/Alert). On the first EMA each day, participants also rated anticipated risky situations, stressful events, and the likelihood that they would drink alcohol in the next week (i.e., abstinence self-efficacy).

### Individual characteristics

We collected self-report information about demographics (age, sex at birth, race, ethnicity, education, marital status, employment, and income) and clinical characteristics (AUD milestones, number of quit attempts, lifetime AUD treatment history, lifetime receipt of AUD medication, DSM-5 AUD symptom count, current drug use [32], and presence of psychological symptoms [31]) to characterize our sample. DSM-5 AUD symptom count and presence of psychological symptoms were also used to determine eligibility. Demographics were included as features in our models. A subset of these variables (sex at birth, race, ethnicity, and income) were used for model fairness analyses, as they have documented disparities in treatment access and outcomes. As part of the aims of the parent project, we collected many other trait and state measures throughout the study. A complete list of all measures can be found on our study’s OSF page (https://osf.io/xta67/).

### Data analytic strategy

Data preprocessing, modeling, and Bayesian analyses were done in R (version 4.4.2) using the tidymodels ecosystem [33–35]. Models were trained and evaluated using high-throughput computing resources provided by the University of Wisconsin Center for High Throughput Computing [36].

### Predictions

**Fig 1:**
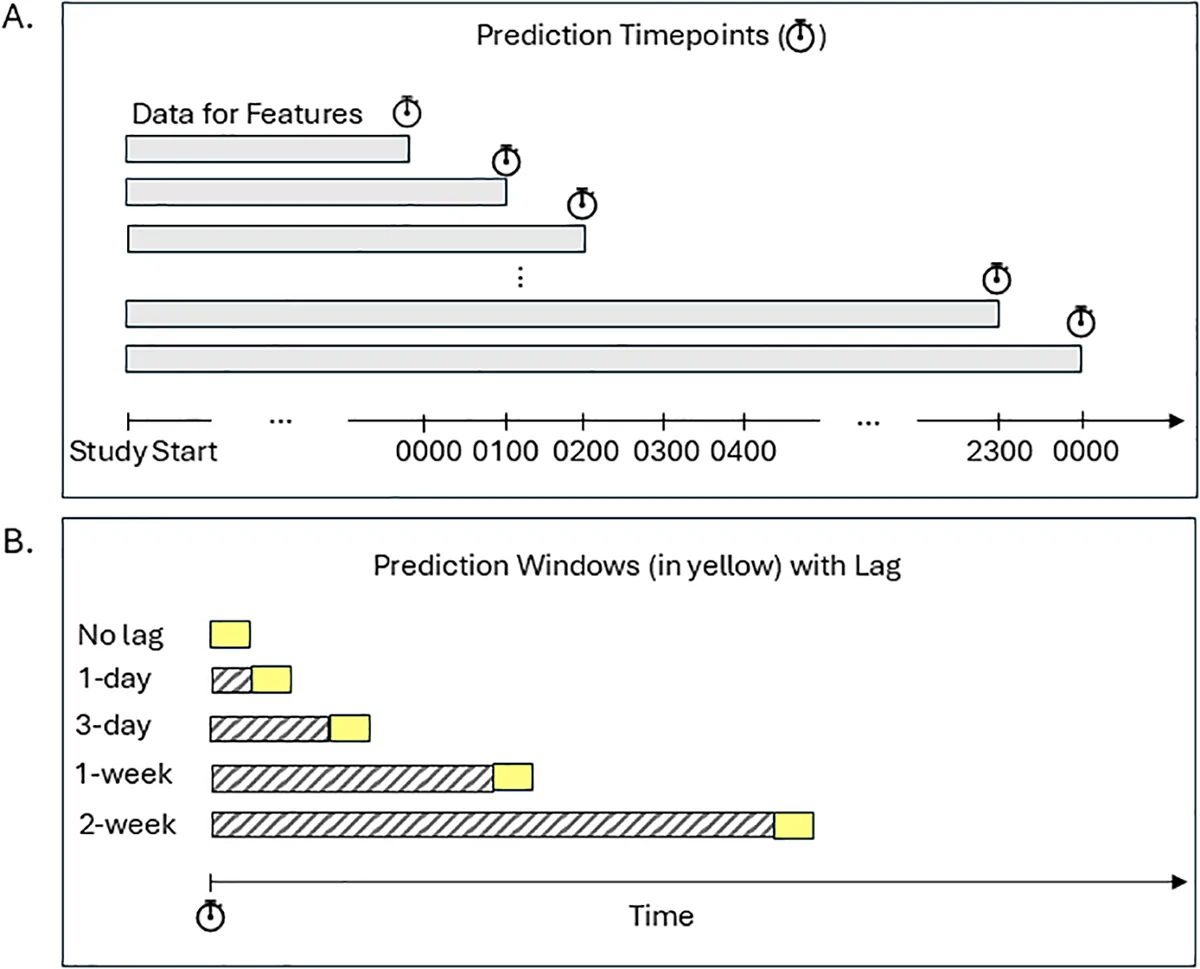
Prediction Timepoints and Prediction Windows. Panel A shows the prediction timepoints at which our model calculated a predicted probability of a lapse. All available data up until, but not including, the prediction timepoint was used to generate these predictions. Features were created for varying feature scoring epochs before the prediction timepoint (i.e., 12, 24, 48, 72, and 168 hours). Prediction timepoints were updated hourly. Panel B shows how the prediction window (i.e., window in which a lapse might occur) rolls forward hour-by-hour with the prediction timepoint. The prediction window width for all models was 24 hours. Additionally, there were five possible lag times between the prediction timepoint and start of the prediction window. A prediction window either started immediately after the prediction timepoint (no lag) or was lagged by 1 day, 3 days, 1 week, or 2 weeks.

A prediction timepoint (Fig 1, Panel A) is the hour at which our model calculates a predicted probability of a lapse within a future 24-hour prediction window for any specific individual. We calculated features from all available EMAs up until, but not including, the prediction timepoint. The first prediction timepoint for each participant was 24 hours from midnight on their study start date. This ensured at least 24 hours of past EMAs were available. Subsequent prediction timepoints for each participant repeatedly rolled forward hour-by-hour until the end of their study participation.

The prediction window (Fig 1, Panel B) spans a period of time in which a lapse might occur. The prediction window width for all models was 24 hours (i.e., models predicted the probability of a lapse occurring within a specific 24-hour period). Prediction windows rolled forward hour-by-hour with the prediction timepoint. However, there were five possible *lag times* between the prediction timepoint and start of the associated prediction window. A prediction window either started immediately after the prediction time point (no lag) or was lagged by 1 day, 3 days, 1 week, or 2 weeks.

Given this structure, our models provided hour-by-hour predicted probabilities of an alcohol lapse in a future 24-hour period (i.e., prediction window). Depending on the model, that prediction window might start immediately after the prediction timepoint (no lag) or it might be lagged to start 1 day, 3 days, 1 week, or 2 weeks after the prediction timepoint. For example, at midnight on the 30th day of participation, features would be calculated from the past 30 days of EMAs. Separate models would predict the probability of lapse for 24-hour prediction windows that start at midnight that day or start 1 day, 3 days, 1 week or 2 weeks after midnight on day 30.

### Labels

The start and end dates and times of past drinking episodes were reported on the first EMA item. A prediction window was labeled “lapse” if the start date/hour of any drinking episode fell within that window. A window was labeled “no lapse” if no alcohol use occurred within that window + /- 24 hours. If no alcohol use occurred within the window but did occur within 24 hours of the start or end of the window, the window was excluded. We used this conservative 24-hour fence for labeling windows as no lapse (vs. excluded) to increase the fidelity of these labels. Given that most windows were labeled no lapse, and the outcome was highly unbalanced, it was not problematic to exclude some no lapse events to further increase confidence in those labels.

This method produced totals of: 274,179 labels for the baseline (no lag) model (7.7% lapse); 270,911 labels for the 1-day lagged model (7.7% lapse); 264,362 labels for the 3-day lagged model (7.6% lapse); 251,458 labels for the 1-week lagged model (7.6% lapse); and 228,420 labels for the 2-week lagged model (7.6% lapse).

### Feature engineering

Features were calculated using only data collected prior to each prediction timepoint to ensure our models were making true future predictions. For the no lag model, the prediction timepoint was at the start of prediction window, so all data prior to the start of the prediction window were included. For the lagged models, the prediction timepoint was 1 day, 3 days, 1 week, or 2 weeks prior to the start of the prediction window, so the last EMA data used for feature engineering were collected 1 day, 3 days, 1 week, or 2 weeks prior to the start of the prediction window.

A total of 285 features were derived from three data sources:

- 1. Prediction window: We dummy-coded features for day of the week at the start of the prediction window.
- 2. Demographics: We created quantitative features for age (in years) and personal income (in dollars), and dummy-coded features for sex at birth (male vs. female), race/ethnicity (non-Hispanic White vs. non-White and/or Hispanic), marital status (married vs. not married vs. other), education (high school or less vs. some college vs. college degree), and employment status (employed vs. unemployed).
- 3. Previous EMA responses: We calculated raw and difference features in varying feature scoring epochs (i.e., 12, 24, 48, 72, and 168 hours) before the prediction timepoint for all EMA items, except the alcohol use question. Raw features included min, max, median, and most recent scores for each EMA item across all EMAs in each epoch for a given participant. We calculated difference features by subtracting the participant’s mean value (using all available data prior to the prediction window) from the associated raw feature value to capture participant-level changes from baseline.
  We calculated raw and difference rate features for past use from reported lapses on the EMA. Raw past use features were generated by dividing the total number of previously reported lapses within a feature scoring epoch by the duration of that epoch. For difference past use rate features, we subtracted the baseline past use rate for that participant (i.e., total number of lapses while on study divided by total hours on study before the prediction window) from their associated raw past use rate. We also calculated raw and difference rate features for completed EMAs (i.e., number of full EMA surveys that were completed in a feature scoring epoch divided by the duration of that epoch).

Features had missing values if the participant did not respond to the relevant EMA question during the associated scoring epoch. The proportion of missing values across features and models was low (median = .02, range = 0 −  .13). We imputed missing data using median imputation for numeric features and mode imputation for nominal features. We selected coarse median/mode methods for handling missing data due to the computational costs associated with more advanced forms of imputation (e.g., KNN imputation, multiple imputation). Importantly, our imputation calculations are done using only held-in data and can be applied to any new observation.

Other generic feature engineering steps included removing zero and near-zero variance features as determined from held-in data. A sample feature engineering script (i.e., tidymodels recipe) containing all feature engineering steps is available on our OSF study page.

### Model configurations

We trained and evaluated five separate classification models: one baseline (no lag) model and one model for 1-day, 3-day, 1-week, and 2-week lagged predictions. We considered four well-established statistical algorithms (elastic net, XGBoost, regularized discriminant analysis, and single layer neural networks) that vary across characteristics expected to affect model performance (e.g., flexibility, complexity, handling higher-order interactions natively) [37]. Candidate model configurations differed across sensible values for key hyperparameters. Configurations also differed on outcome resampling method (i.e., no resampling and up-sampling and down-sampling of the outcome using majority/no lapse to minority/lapse ratios ranging from 5:1–1:1).

### Cross-validation

We used nested cross-validation for model training, selection, and evaluation. Nested cross-validation involves using two nested cross-validation loops to produce unbiased estimates of how well a model performs on new data from new individuals. The inner loop holds out data from model training for model selection (i.e., validation sets) and the outer loop holds out new data not previously seen by the respective inner loops (for training or validation) for model evaluation (i.e., test sets).

We used 2 repeats of 5-fold cross-validation for the inner loops and 6 repeats of 5-fold cross-validation for the outer loop. Folds were stratified so that all folds contained comparable proportions of individuals who lapsed frequently (i.e., 10+ times) and participants were grouped so that all of their data were always in the held-in or held-out fold (validation/test) for a split, but never in both.

Our performance metric was area under the receiver operating characteristic curve (auROC). auROC indexes the probability that the model will predict a higher score for a randomly selected positive case (lapse) relative to a randomly selected negative case (no lapse). The best model configurations were selected using median auROC across the 10 validation sets. Final performance of those best model configurations were evaluated using median auROC across the 30 test sets.

We also report median sensitivity, specificity, and balanced accuracy at a single decision threshold (identified using Youden’s index) in Table A in S1 Appendix. However, we caution against using classification in this intended application. A decision threshold is only necessary when a “decision” about whether to intervene must be made. We intend for our model’s predicted probabilities to be used to provide risk-relevant information to an individual irregardless of their predicted risk level. We believe that assigning a class label of lapse versus no lapse would be less clinically useful (compared to these full-range probability scores) and may even be potentially iatrogenic. Moreover, the optimal decision threshold is likely to vary substantially depending on the clinical context and the relative costs of misclassification.

### Bayesian model

We used a Bayesian hierarchical generalized linear model to estimate the posterior probability distributions and 95% Bayesian credible intervals (CIs) from the 30 held-out test sets for our five best models. Following recommendations from the rstanarm team and others [38,39], we used the rstanarm default autoscaled, weakly informative, data-dependent priors that take into account the order of magnitude of the variables to provide some regularization to stabilize computation and avoid over-fitting. Priors were set as follows: Residual SD ~ exponential(2.6); intercept (centered predictors) ~ normal(2, 0.98); fixed effects ~ normal(0, 2.43); covariance ~ decov(1, 1, 1, 1). We set two random intercepts to account for our resampling method: one for the repeat, and another for the fold nested within repeat. We specified two sets of pre-registered contrasts for model comparisons. The first set compared each lagged model to the baseline no lag model (1-day lag vs. no lag, 3-day lag vs. no lag, 1-week lag vs. no lag, 2-week lag vs. no lag). The second set compared adjacently lagged models (3-day lag vs. 1-day lag, 1-week lag vs. 3-day lag, 2-week lag vs. 1-week lag). auROCs were transformed using the logit function and regressed as a function of model contrast.

From the Bayesian model we obtained the posterior distribution (transformed back from logit) and Bayesian CIs for auROCs for all five models. To evaluate our models’ overall performance, we report the median posterior probability for auROC and Bayesian CIs. This represents our best estimate for the magnitude of the auROC parameter for each model. If the CIs do not contain  .5 (chance performance), this provides strong evidence (>  .95 probability) that our model is capturing signal in the data.

We then conducted Bayesian model comparisons using our two sets of contrasts – baseline and adjacent lags. For both model comparisons, we determined the probability that the models’ performances differed systematically from each other. We also report the precise posterior probability for the difference in auROCs and the 95% Bayesian CIs.

### Fairness analysis

Using the same 30 held-out test sets, we calculated the median posterior probability and 95% Bayesian CI for auROC for each model separately by race/ethnicity (non-White and/or Hispanic vs. non-Hispanic White), income (below poverty line vs. above poverty line), and sex at birth (female vs. male). We used course race/ethnicity groupings due to the limited diversity of these demographics in our sample. The poverty cutoff was defined from the 2024 federal poverty line for the 48 contiguous United States. Participants at or below $15,060 annual income were categorized as below poverty. We conducted Bayesian group comparisons to assess the likelihood that each model performs differently by group. We summarize the differences in posterior probabilities for auROC across models. Individual Bayesian fairness contrasts for all five models are available in Table B in S1 Appendix.

### Model calibration

To further characterize and understand our models, we used our inner resampling procedure (2 repeats of 5-fold cross validation grouped on participant) on the full data set to select a single best model configuration for each classification model (no lag, 1-day, 3-day, 1-week, and 2-week lag). The final configuration selected for each model represents the most reliable and robust configuration for deployment.

The best model configuration for each classification model was fit on the full data set. We fit this configuration using single 5-fold cross-validation. This method allowed us to obtain a single predicted probability for each observation, while still using separate data for model training and prediction. We calibrated our probabilities using Platt scaling [40]. We calculated Brier scores to assess the accuracy of our raw and calibrated probabilities and provide calibration plots.

### Feature importance

We used the same single 5-fold cross-validation procedure to calculate raw Shapley values for observations in our held-out folds. Raw Shapley values index the importance of any feature (or set/category of features as described below) to any single prediction for a specific observation (i.e., for a specific 24-hour window for a specific participant), which indicates the “local importance” of that feature [22]. More precisely, the magnitude of the raw Shapley value for any feature indicates how much the feature score for that observation adjusted the prediction (in log-odds units) for that observation relative to the mean prediction across all observations. Positive Shapley values indicate that the feature score increased the prediction for that observation and negative values indicate that the feature score decreased the prediction.

Raw Shapley values are additive across features for an observation, with their sum across features equal to the total adjustment of the predicted value for that observation vs. the mean predicted value across all observations. This property allows raw Shapley values to be added together across features within a category to index the importance of that feature category. We created feature categories by summing raw Shapley values for all features associated with specific EMA items. In three instances, we combined features across two similar EMA items (i.e., past and anticipated risky situations, past and anticipated stressful events, and affective valence and arousal) to yield seven feature categories for distinct constructs assessed by the original 10 EMA items. Specifically, we calculated Shapley values for past use, craving, affective state, past/anticipated risky situations, past/anticipated stressful events, past pleasant events, and abstinence self-efficacy.

We summarized feature importance in two ways. First, we used a traditional approach in which we calculated the global importance of each feature category (mean absolute Shapley value). Global feature importance describes how important a feature is, on average, across all observations from all participants. A large mean absolute Shapley value indicates that a feature category makes substantial contributions to predictions across the dataset. Second, we characterized local feature importance by calculating the proportion of observations in which each feature category had the highest Shapley value. This approach summarizes how frequently a feature category is the most influential contributor to individual predictions. We provided a descriptive plot of the relative ranking of feature categories by their global and local feature importance for the no lag and 2-week lagged models.

## Results

### Demographic and lapse characteristics

Table 1 provides a detailed breakdown of the demographic and clinical characteristics of our sample (N = 151).

**Table 1:** Demographic and clinical characteristics.

Age			41	11.9	21-72
Sex at Birth					
Female	74	49.0			
Male	77	51.0			
Race					
American Indian/Alaska Native	3	2.0			
Asian	2	1.3			
Black/African American	8	5.3			
White/Caucasian	131	86.8			
Other/Multiracial	7	4.6			
Hispanic, Latino, or Spanish origin					
Yes	4	2.6			
No	147	97.4			
Education					
Less than high school or GED degree	1	0.7			
High school or GED	14	9.3			
Some college	41	27.2			
2-Year degree	14	9.3			
College degree	58	38.4			
Advanced degree	23	15.2			
Employment					
Employed full-time	72	47.7			
Employed part-time	26	17.2			
Unemployed	53	35.1			
Personal Income			$34,298	$31,807	$0-200,000
Marital Status					
Never married	67	44.4			
Married	32	21.2			
Divorced, separated or widowed	52	34.4			
DSM-5 Alcohol Use Disorder Symptom Count			8.9	1.9	4-11
Alcohol Use Disorder Milestones					
Age of first drink			14.6	2.9	6-24
Age of regular drinking			19.5	6.6	11-56
Age at which drinking became problematic			27.8	9.6	15-60
Age of first quit attempt			31.5	10.4	15-65
Number of Quit Attempts*			5.5	5.8	0-30
Lifetime History of Treatment (Can choose more than 1)					
Long-term residential (6+ months)	8	5.3			
Short-term residential (< 6 months)	49	32.5			
Outpatient	74	49.0			
Individual counseling	97	64.2			
Group counseling	62	41.1			
Alcoholics Anonymous/Narcotics Anonymous	93	61.6			
Other	40	26.5			
Received Medication for Alcohol Use Disorder					
Yes	59	39.1			
No	92	60.9			
Current (Past 3 Month) Drug Use					
Tobacco products (cigarettes, chewing tobacco, cigars, etc.)	84	55.6			
Cannabis (marijuana, pot, grass, hash, etc.)	66	43.7			
Cocaine (coke, crack, etc.)	18	11.9			
Amphetamine type stimulants (speed, diet pills, ecstasy, etc.)	15	9.9			
Inhalants (nitrous, glue, petrol, paint thinner, etc.)	3	2.0			
Sedatives or sleeping pills (Valium, Serepax, Rohypnol, etc.)	22	14.6			
Hallucinogens (LSD, acid, mushrooms, PCP, Special K, etc.)	14	9.3			
Opioids (heroin, morphine, methadone, codeine, etc.)	16	10.6			
Reported 1+ Lapse During Study Period					
Yes	84	55.6			
No	67	44.4			
Number of reported lapses			6.8	12	0-75

N = 151.

*Two participants reported 100 or more quit attempts. We removed these outliers prior to calculating the mean (M), standard deviation (SD), and range.

### Model evaluation

**Fig 2:**
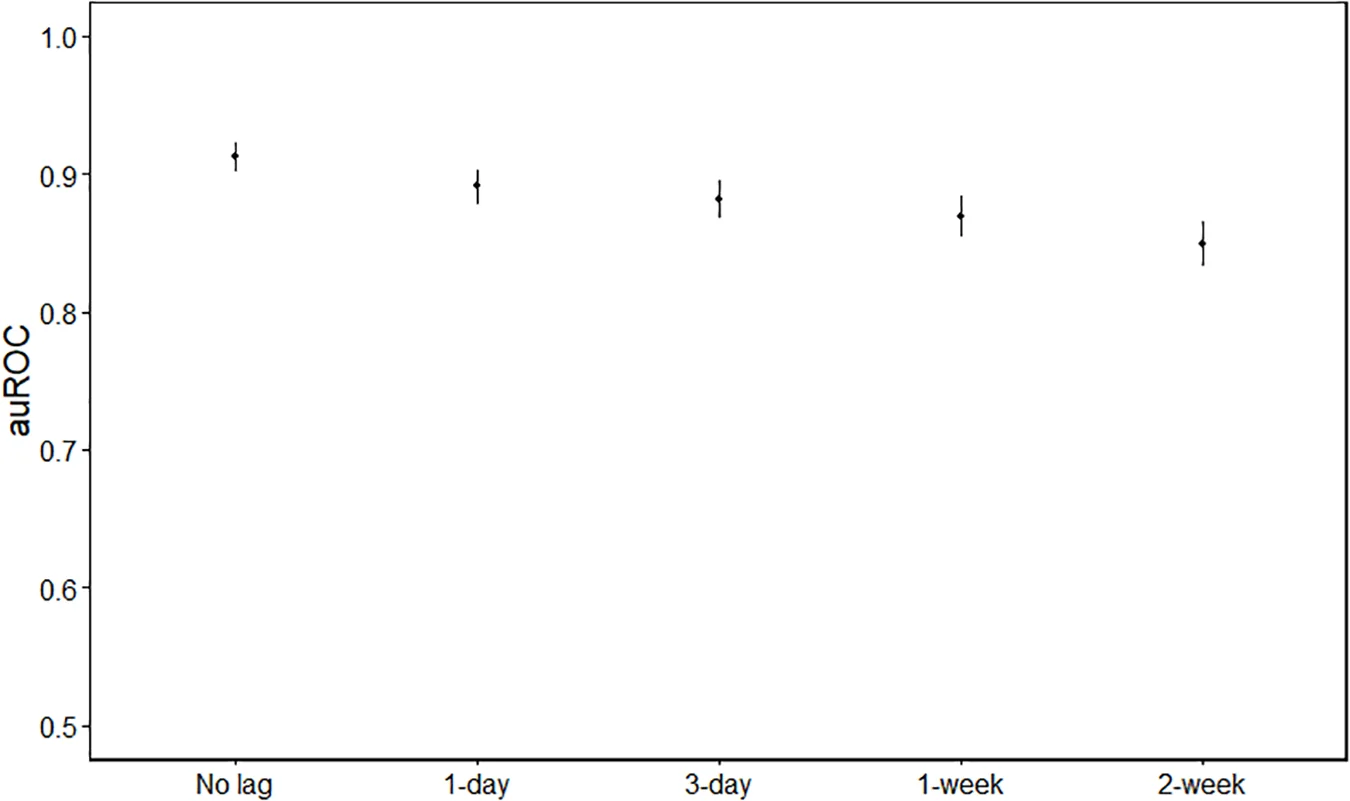
Posterior Probabilities for Area Under the Receiver Operating Characteristic Curve (auROC) by Model (No lag, 1-day, 3-day, 1-week, 2-week). auROC ranges from  .5 (chance performance) to 1 (perfect performance). Median posterior probabilities over 12,000 posterior samples (4 chains × 3,000 samples) are depicted as points. Vertical lines represent the 95% Bayesian credible intervals for the posterior distribution.

Fig 2 presents the median posterior probability for auROC and 95% Bayesian CI for each model (no lag, 1-day, 3-day, 1-week, and 2-week lag). The median auROCs were 0.91 (no lag), 0.89 (1-day lag), 0.88 (3-day lag), 0.87 (1-week lag), and 0.85 (2-week lag). These values represent our best estimates for the magnitude of the auROC parameter for each model. The 95% Bayesian CI for the auROCs for these models were relatively narrow and did not contain 0.5: no lag [0.90–0.92], 1-day lag [0.88–0.90], 3-day lag [0.87–0.90], 1-week lag [0.85–0.88], 2-week lag [0.83–0.87].

We also evaluated how well our models predicted first lapses compared to later lapses (Figure A in S1 Appendix). Overall, performance was worse for first lapses than for later lapses, remaining relatively stable across lag times (median auROCs of 0.78–0.79). In contrast, the model performed very well for later lapses (median auROCs of 0.87–0.94). Performance for later lapses also showed a moderate decline as lag time increased, mirroring the pattern observed in overall model performance.

### Model comparisons

Table 2 presents the median difference in auROC, 95% Bayesian CI, and posterior probability that that the auROC difference was smaller than 0 for all baseline and adjacent lag contrasts. Median auROC differences less than 0 indicate the more lagged model, on average, performed worse than the more immediate model (e.g., 1-day lag – no lag, 3-day lag – 1-day lag). There was strong evidence (probabilities = 1) that the lagged models performed worse than the baseline (no lag) model, with average drops in auROC ranging from 0.02–0.06, and the previous adjacent lagged model, with average drops in auROC ranging from 0.01–0.02.

**Table 2:** Median difference in auROC, 95% Bayesian Credible Interval (CI), and posterior probability that the auROC difference was smaller than 0 for all baseline and adjacent lag contrasts.

Contrast	Median	Bayesian CI	Probability
Baseline Contrasts			
1-day vs. No lag	−0.021	[-0.025, -0.017]	1
3-day vs. No lag	−0.03	[-0.035, -0.025]	1
1-week vs. No lag	−0.042	[-0.048, -0.037]	1
2-week vs. No lag	−0.062	[-0.07, -0.056]	1
Adjacent Contrasts			
3-day vs. 1-day	−0.009	[-0.013, -0.005]	1
1-week vs. 3-day	−0.012	[-0.017, -0.008]	1
2-week vs. 1-week	−0.02	[-0.026, -0.015]	1

Median auROC differences less than 0 indicate the more lagged model, on average, performed worse than the more immediate model (e.g., 1-day lag – no lag, 3-day lag – 1-day lag). Bayesian CI represents the range of values where there is a 95% probability that the true auROC difference lies within that range. Probability indicates the posterior probability that this difference is smaller than 0 (i.e., the models are performing differently).

### Fairness analysis

Table 3 presents the median difference in auROC, 95% Bayesian CI, and posterior probability that the auROC difference was smaller than 0 for the three fairness contrasts: race/ethnicity (non-White and/or Hispanic; *N* = 20 vs. Non-Hispanic White; *N* = 131), sex at birth (female; *N* = 74 vs. male; *N* = 77), and income (below poverty line; *N* = 49 vs. above poverty line; *N* = 102). Median auROC differences less than 0 indicate the model, on average, performed worse for the non-advantaged group (female, non-White and/or Hispanic, below poverty line) compared to the advantaged group (male, non-Hispanic White, and above poverty line). In Table 3 we present fairness analyses for our baseline model (no lag) and for our longest lagged model (2-week lag), as this is likely the most clinically useful lagged model for providing advanced warning of lapse risk. Fairness analyses for all five models are available in Table B in S1 Appendix.

**Table 3:** Median difference in auROC, 95% Bayesian Credible Interval (CI), and posterior probability that the auroc difference was smaller than 0 for fairness contrasts for the no lag and 2-week lagged models.

Contrast	Median	Bayesian CI	Probability
Fairness Contrasts (No Lag)			
female vs. male	−0.043	[-0.059, -0.028]	1
non-White and/or Hispanic vs. non- Hispanic White	−0.131	[-0.222, -0.057]	0.999
below poverty line vs. above poverty line	−0.012	[-0.033, 0.007]	0.848
Fairness Contrasts (2-week Lag)			
female vs. male	−0.098	[-0.125, -0.073]	1
non-White and/or Hispanic vs. non- Hispanic White	−0.13	[-0.208, -0.058]	0.998
below poverty line vs. above poverty line	−0.039	[-0.073, -0.008]	0.98

Median auROC differences less than 0 indicate the model, on average, performed worse for the disadvantaged group (female, non-White and/or Hispanic, income below poverty line) compared to the advantaged group (male, non-Hispanic White, income above poverty line). Bayesian CI represents the range of values where there is a 95% probability that the true auROC difference lies within that range. Probability indicates the posterior probability that this difference is smaller than 0 (i.e., the models are performing differently for fairness subgroups).

There was strong evidence (probabilities > .84) that our models performed worse for the non-advantaged groups compared to the advantaged groups. On average, across all five models, there was a median decrease in auROC of 0.13 (range 0.13–0.17) for participants who were non-White and/or Hispanic compared to participants who were non-Hispanic White. On average, across all five models, there was a median decrease in auROC of 0.05 (range 0.04–0.10) for female participants compared to male participants. On average, across all five models, there was a median decrease in auROC of 0.02 (range 0.01–0.04) for participants below the federal poverty line compared to participants above the federal poverty line.

The proportion of positive lapse labels over all labels (lapse and no lapse) for each demographic subgroup were relatively consistent across groups: race/ethnicity (6%, non-White and/or Hispanic vs. 8%, non-Hispanic White), income (12%, below poverty line vs. 7%, above poverty line), sex at birth (9%, female vs. 7%, male).

### Model calibration

The raw probabilities produced by our final models were not well calibrated. Consequently, we used Platt scaling to improve calibration. Brier scores range from 0 (perfect accuracy) to 1 (perfect inaccuracy). Platt scaling showed excellent improvement to the no lag model with a Brier score of  .043. Calibration also improved probability accuracy for the 2-week lagged model with a Brier score of  .063. For comparison, raw probability scores yielded Brier scores of  .071 and  .077 for the no lag and 2-week lagged models, respectively. A table of Brier scores for all five models is available in Table C in S1 Appendix.

**Fig 3:**
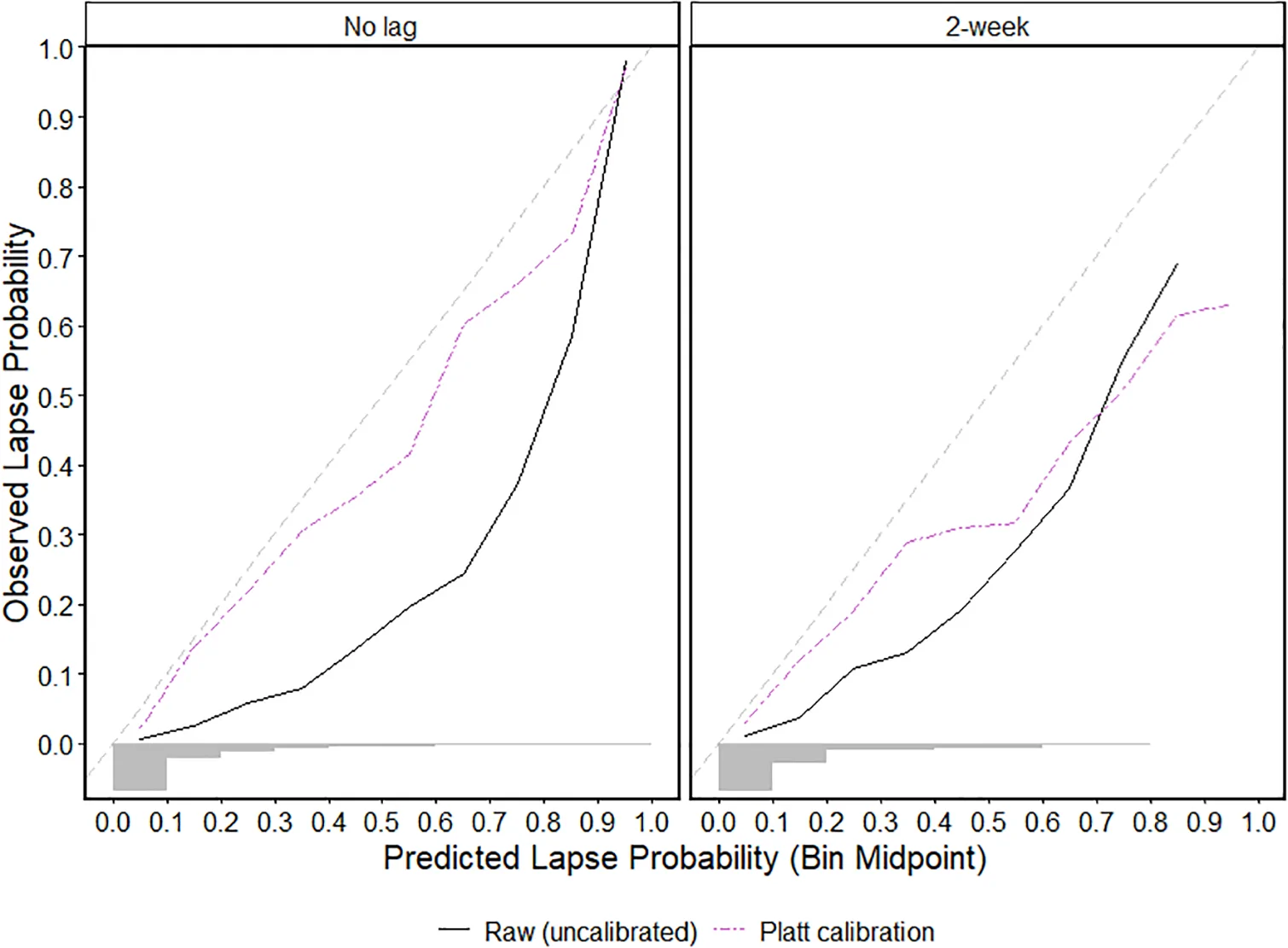
Calibration Plots of Raw and Calibrated Lapse Probabilities for the Baseline (No Lag) and 2-week Lagged Models. Predicted probabilities (x-axis) are binned into deciles. Observed lapse probability (y-axis) represents the proportion of actual lapses observed in each bin. The dashed diagonal represents perfect calibration. Points below the line indicate overestimation and points above the line indicate underestimation. Raw probabilities are depicted as solid black curves. Platt calibrated probabilities are depicted as pink dashed curves. The grey histogram along the bottom of the plot represents the proportion of raw probabilities in each bin.

Fig 3 shows the calibration plots for the raw and calibrated probabilities for the no lag and 2-week lagged model. It also includes a histogram of raw probabilities that demonstrates our models produced variable predicted probabilities, spanning nearly the entire 0–1 range. Calibration plots for all 5 models are available in Figure B in S1 Appendix.

### Feature importance

Global feature importance is an indicator of how important a feature category was to the model’s predictions, on average (i.e., across all participants and all observations). The top globally important feature category (i.e., highest mean |Shapley value|) for all models was past use. Future efficacy was a strong predictor for more immediate model predictions (i.e., no lag), but its importance diminished as lag time increased. On the other hand, as lag time increased, past/future risky situations increased in importance. Craving was consistently important in magnitude across all models. A plot of global feature importance as a function of lag time is available in Figure C in S1 Appendix. These findings were also consistent across demographic subgroups (plots of global feature importance by demographic subgroup are available for the no lag and 2-week lagged models in Figure D in S1 Appendix).

**Fig 4:**
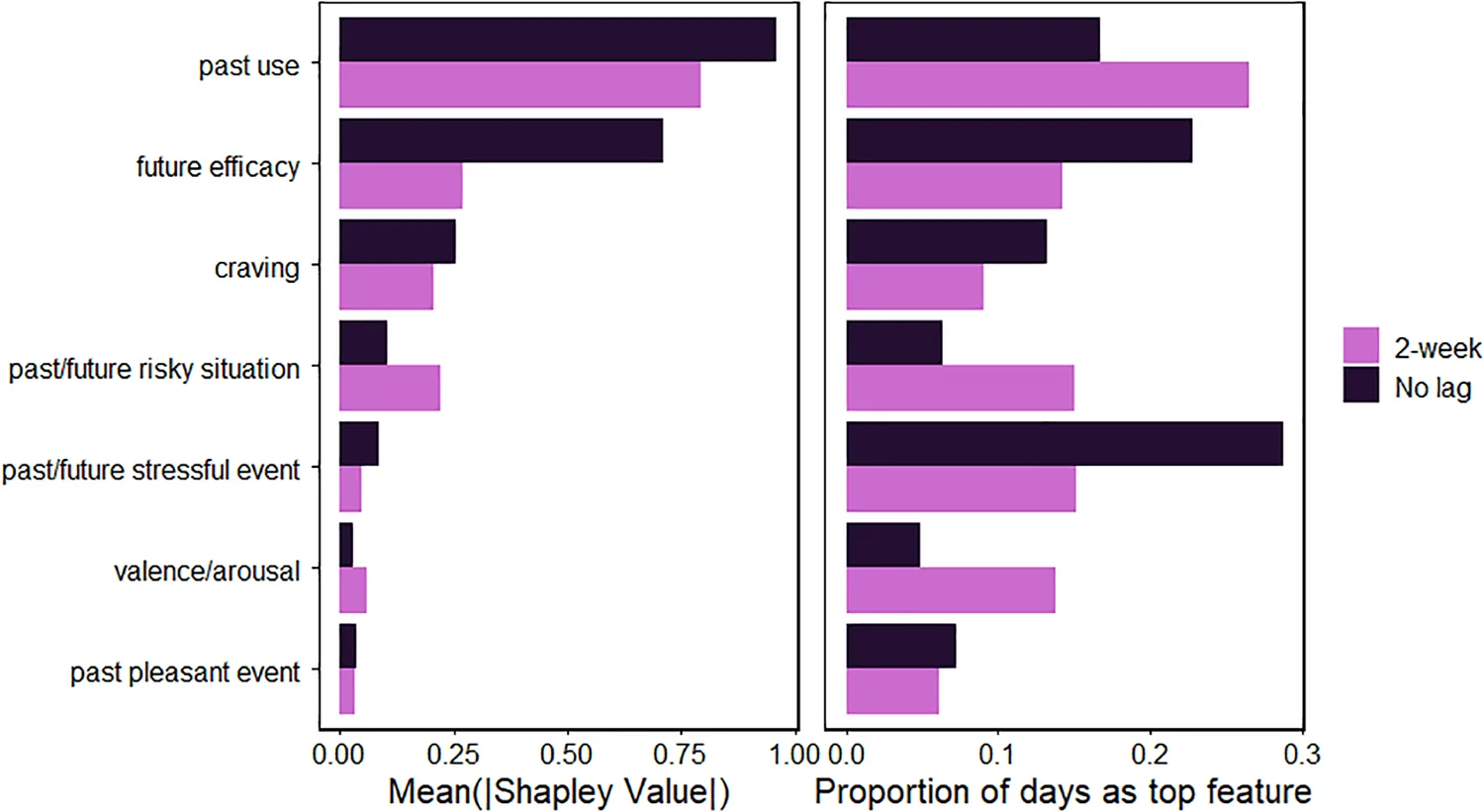
Global (mean |Shapley value|) and Local (proportion of days as top feature) Feature Importance for Feature Categories for the No Lag and 2-week Lagged Models. Feature categories are ordered by their aggregate global importance. The importance of each feature category for each model is displayed separately by color.

Local importance describes how important a feature category was for individual predictions. In the immediate model, past/future self-reported stressful events emerged as the most important feature category on most days, despite having a relatively small influence on predictions at the global level. Future efficacy was the second most important feature category, followed by past use. In the 2-week lagged model, past use remained the most important feature category (i.e., it produced the highest Shapley value for the greatest number of individuals on the most days). Past/future risky situations also remained among the top feature categories. However, valence/arousal additionally emerged as a competing top feature category (a pattern not observed in the immediate model). Fig 4 shows the relative global and local ranking of feature categories for the no lag and 2-week lagged models.

## Discussion

### Model evaluation and comparisons

All the models that we evaluated performed exceptionally well. The no lag model, which predicts the probability of an immediate (i.e., within 24 hours) lapse back to alcohol use, had a  .91 median posterior probability for auROC. Our 2-week lagged model, which made the most distal predictions, had a  .85 median posterior probability for auROC, suggesting lagged models can be used to shift a 24-hour prediction window meaningfully into the future.

Across models (no lag, 1 day, 3 days, 1 week, and 2 weeks), model performance systematically decreased as models predicted further into the future. All lagged models had lower performance compared to the no lag baseline model and to the preceding adjacent lag model. This is unsurprising given what we know about prediction and substance use. Many important relapse risk factors are fluctuating processes that can first emerge and/or change day-by-day, if not more frequently. As lag time increases, features become less proximal to the start of the prediction window. Still, we wish to emphasize that our lowest auROC (.85) is still quite good, and the benefit of advanced notice (i.e., 2 weeks) likely outweighs the modest cost to model performance.

Our models performed better at predicting lapses once a lapse had already occurred. Lower performance in predicting an individual’s first lapse is not necessarily problematic. A single lapse does not constitute a relapse but instead represents a period of vulnerability, during which an individual may either continue to lapse (and potentially relapse, or return to prior levels of hazardous use) or learn from the experience and re-engage with their recovery goals. Given that this critical window often follows the initial lapse, we are confident that our models can still be used to provide timely recovery support.

Collectively, these results suggest we can achieve clinically meaningful performance up to two weeks out. Our rigorous resampling methods (grouped, nested, k-fold cross-validation) make us confident that these are valid estimates of how our models would perform with new individuals. Furthermore, it should be noted that both the no lag and 2-week lagged models can be combined in a complementary fashion that allows both for highly accurate immediate lapse prediction and advanced warning about future lapse risk.

### Model fairness

In recent years, the machine learning field has begun to recognize the need to evaluate model fairness when algorithms are used to inform important decisions (e.g., healthcare services offered, eligibility for loans, early parole). Algorithms that perform favorably for only majority group members may exacerbate existing disparities in access to resources and important clinical outcomes [41]. In this study, we assessed model fairness by comparing model performance across important subgroups with known disparities in substance use treatment access and/or outcomes – race/ethnicity (non-White and/or Hispanic vs. non-Hispanic White), income (below poverty line vs. above poverty line), and sex at birth (female vs. male).

All models performed worse for people who were non-White and/or Hispanic, and for people who had an income below the poverty line. The lack of diversity in our training data was likely a key contributor to the poorer model performance in these subgroups. Participants of color were severely underrepresented in our training data (*N* = 20, 13%). Individuals below the poverty line were also underrepresented, though to a lesser degree (*N* = 49, 32%). Of course, these model comparisons should be considered preliminary and interpreted cautiously because the low sample sizes in some groups (e.g., non-White and/or Hispanic) may reduce the statistical validity of the analyses. Nonetheless, these preliminary results regarding model fairness should motivate further efforts to evaluate and address differences in model performance across groups that differ in SUD treatment access/outcome disparities.

An obvious solution to this problem involves intentional recruitment for diversity in training data when developing prediction models. For example, we are now working to increase the racial, ethnic, and income diversity of our training data for alcohol lapse prediction while simultaneously optimizing feedback from these models for implementation purposes [25]. In a separate project, we developed a national recruitment method that enabled us to recruit for racial, ethnic and income diversity while also focusing on much needed diversity across geographic location (e.g., rural vs. urban; [20]). We expect geographic diversity in the training data may also be crucial to develop fair models because the features that predict lapse in urban and suburban settings may differ from those that predict lapse in rural environments. If rural participants are not used to train models, the implementation of these models may compound existing disparities in SUD treatment in these communities [42,43].

Future research can also explore potential computational solutions to mitigate performance disparities that emerge when subgroups are poorly represented in available training data. For example, training data from under-represented subgroups could be up-sampled (e.g., using the synthetic minority oversampling technique), or the cost functions used by the learning algorithms could be adjusted to differentially weigh prediction errors based on participant characteristics. In another vein, modeling approaches that yield idiographic, person-specific models [44–47] may reduce performance disparities across subgroups. For example, we have begun to develop state space models whose parameters can be initialized with priors derived from existing training data but then adjusted over time to fit patterns present within a specific individual’s time-series [48]. Such models may mitigate issues of unfairness to a large degree because they will weigh the individual’s own data more heavily than group level estimates over time as more data accrue.

Of note, problems with model fairness can emerge even when subgroups are well-represented in the training data. Our models performed less well for women compared to men despite the fact that women were well-represented in the training data (*N* = 74, 49%). Instead, this differential performance may have resulted from more fundamental problems with the features available to the model. We chose our EMA items using domain expertise from decades of research on the factors that predict relapse. However, prior to the 1993 National Institute of Health Revitalization Act [49] that mandated the inclusion of minorities and women in research, women were mostly excluded from substance use treatment research due to their childbearing potential [50]. As a result, it is possible that our theories about the causes and contributors to relapse are biased toward constructs that are more relevant for men than women. If true, features derived from EMA items that tap these constructs would be expected to under-perform when predicting lapses for women. More research may be needed to identify relapse risk factors for women (e.g., interpersonal relationship problems [51], hormonal changes [52]), and other groups under-represented in the literature before we can fully address these performance disparities.

In the meantime, data-driven (bottom-up) approaches can be used to engineer high-dimensional feature sets that are not explicitly grounded in existing, and potentially biased, theories. For example, we have begun to explore the application of natural language processing techniques (e.g., LIWC; topic modeling; BERT [53–55]) to text messages and other social media activity by our participants to engineer features that may predict future lapses. Such features may or may not align with existing theories about relapse, but because they are anchored to participants’ own words, they may serve as reliable indicators of lapse risk for certain individuals, particularly when used within learning algorithms that employ feature selection, regularization, or other techniques to address the bias-variance trade-off with high-dimensional feature sets. Furthermore, emerging techniques for interpreting machine learning models [56] can be applied to models that perform well to bootstrap the identification of new lapse risk constructs based on these novel features.

Beyond issues of training data representation and lacunae or outright biases in our theories, it is also true that historically marginalized groups that have experienced systemic racism, exclusion, or other stigma around substance use (e.g., societal expectations for women regarding attractiveness, cleanliness and motherhood [57]) may feel less trusting in disclosing substance use [58]. These experiences could prompt some individuals in these subgroups to under-report lapses and/or risk factors, which could also degrade performance and evaluation of our models for these subgroups. We observed relatively comparable percentages of lapses reported among disadvantaged compared to advantaged groups. However, comparable lapse rates do not necessarily confirm comparable reporting accuracy because it is possible that there were systematic differences in lapse rates across groups that were masked by issues of trust.

### Calibration

After applying Platt scaling to our predicted probabilities, our models were generally well calibrated with increasing monotonic relationships between calibrated model output and lapse event rates. Well-calibrated probabilities indicate that the predicted probability aligns closely with the true likelihood of an outcome (i.e., a lapse). Our no lag model had excellent calibration. However, the calibration plots suggest that with a longer lag time of 2 weeks, the model tends to over-predict the likelihood of lapses when predicted probabilities were higher.

This pattern may not necessarily be problematic. Research suggests that people often struggle to interpret probabilistic feedback, especially when it’s provided in raw numerical form [59–61]. As a result, it may be more effective to communicate risk using coarser categories (e.g., low, medium, or high risk) or through relative changes in risk (e.g., “Your risk of lapse is higher this week compared to last week”). These forms of feedback may be less sensitive to small miscalibrations at the extremes as long as the relationship between predicted probabilities and the observed event rate is monotonic.

### Feature importance

The relative ordering of top global features remained somewhat consistent across the no lag and 2-week lagged models. Past use was the most important feature in both models in our dataset. This is not surprising given that our outcome was lapse, and past behavior is often the best predictor of future behavior. This finding also supports decades of clinical research on relapse prevention, where lapses (i.e., single instances of goal inconsistent alcohol use) are seen as powerful precursors to relapse (i.e., full return to harmful drinking; [9]). Abstinence self-efficacy emerged as the second most important feature in both models in our dataset, indicating that participants had reasonably accurate insight into their near-term success with maintaining alcohol abstinence. Craving was also an important predictor in both models, suggesting that it may be an important target for intervention to support early recovery efforts.

Several feature categories displayed sizeable differences in global importance by lag time. The importance of abstinence self-efficacy dropped by more than 50% in the 2-week lagged model relative to the no lag model. This may indicate that self-efficacy during early recovery is unstable even across shorter periods of time such that their current self-efficacy does not strongly predict abstinence success even two weeks into the future. In fact, craving and risky situations become as important as self-efficacy when predicting 2-week lagged lapses. It may be that these other experiences are shaping and changing the individual’s self-efficacy rapidly in early recovery. This also suggests that more frequent clinical assessments of self-efficacy as a target for intervention may be needed rather than assuming stability in this construct if initial assessment suggests it is high. Also, our study cannot determine if this differential importance of self-efficacy for immediate vs. lagged lapses persists beyond early recovery (where people may be encouraged to take a “one day at a time” mindset). Self-efficacy may become a more stable predictor of future abstinence success after longer periods of recovery, but our sample was limited to participants in early recovery (<= 8 weeks of abstinence at intake).

Past use was less important for the 2-week lagged model compared to the no lag model. This indicates that the predictive strength of a lapse on the likelihood of subsequent lapses diminishes to some degree over a relatively short period of time. This is good news and reinforces that single lapses do not always mark a return to consistent patterns of frequent, and potentially harmful, alcohol use. Despite this reduction in importance of past use as a predictor of lagged alcohol use, past use did remain the most important category for 2-week lagged lapses. Lapses may provide “teachable moments” that can be used to reinforce recovery motivation, better understand risks, and develop skills to address those risks [11]. Conversely, lapses should not be ignored because they remain strong predictors of further use.

Surprisingly, past and anticipated risky situations were more important in the 2-week lagged vs. no lag model, suggesting that the impact of these situations on lapses back to use may be delayed. It may be that persistent exposure to risks is necessary to undermine an abstinence goal and lead to return to alcohol use. Alternatively or additionally, people may also be better able to anticipate future risky situations (e.g., vacations, anniversaries of significant dates) than future acute stressors or even future self-efficacy. Regardless, the increased importance of risky situations for predicting lagged lapses provides an opportunity to intervene prior to the lapse, particularly if the individual is encouraged to assess future risks and/or makes use of a recovery monitoring prediction model.

We were also surprised that stressful events, pleasant events, and affective state features did not make more important contributions to predictions across models. These constructs are highlighted in numerous theories about addiction and relapse [9,10,62] and represent targets for intervention in many existing treatments [18,63–65]. It may be that their impact is subsumed within other more powerful features (i.e., past use and self-efficacy). However, this seems unlikely given that the methodology underlying Shapley values allows for a fair distribution of importance among the relevant predictive features even when those features are correlated [56]. Alternatively, we may need to more carefully consider the nuanced roles that these constructs play (e.g., within the context of individual coping strategies, social support or environmental factors) in the return to alcohol use during recovery [66].

Our local feature importance approach, where we calculated the proportion of observations for which each feature category had the highest Shapley value, showed that although past use was the most influential feature overall, it was the top feature on only a subset of days (less than 20% of days in the immediate model and less than 30% in the 2-week lagged model). In fact, past alcohol use fell to the third most important feature for the no-lag model. This is not surprising, as lapses were relatively infrequent (7.6–7.7% of observations), and nearly half of participants did not report any lapses during the study.

Our examination of local feature importance also revealed new important features. In both models, past/future stressful events emerged as an important feature. The fact this did not show up as an important global feature could indicate several things. First, it could be that stressful events are important for predicting lapses for a subset of individuals, but not for others. Second, stress may have a strong impact on some high-risk days but little to no effect on most other days. Finally, as mentioned earlier, it is possible that the influence of stress may overlap with related constructs. In all of these situations, the global Shapley value would be diluted, but stress could still emerge as a highly important predictor in specific contexts or for particular individuals. In the 2-week lagged model, valence/arousal emerged as an important feature. This was somewhat surprising, given that these states can fluctuate rapidly. Their importance in the 2-week lagged predictions, but not in the immediate predictions, may suggest that changes in valence and arousal reflect a vulnerability to lapse that accumulates over time, rather than immediately triggering a lapse.

Our local importance analyses highlight a potential limitation in the interpretability of global feature importance. Global feature importance may be biased toward features that exhibit infrequent but very large Shapley values, potentially overstating the importance of predictors that are strongly associated with the outcome but occur in only a small subset of observations. In contrast, individuals may more often benefit from recovery recommendations that focus on managing stress (e.g., guided relaxation), increasing abstinence confidence (e.g., reflecting on recent successes), and coping with risky situations (e.g., trigger mapping).

Finally, this approach showed that while some features have low average importance (across all individuals and observations), they can be highly important on certain days for certain individuals. Thus, this approach more closely aligns with our precision-guided goal of understanding which risk factor is most influential for a specific individual at a specific moment. In the context of recovery, where lapses are important but relatively infrequent events, features other than past use will, in most cases, represent more clinically meaningful targets for intervention.

### Additional limitations and future directions

We believe our lapse prediction models will be most effective when embedded in an RMSS designed to deliver adaptive and personalized continuing care. This system could send daily, weekly, or less frequent messages to users with personalized feedback about their risk of lapse and provide support recommendations tailored to their current recovery needs. This study provides initial support that immediate and lagged prediction models can be trained to high accuracy using EMA for recovery monitoring. Furthermore, locally important features from these models can be used to identify the specific factors that contribute to each lapse risk prediction.

The no lag model can be used to guide individuals to take immediate, actionable steps to maintain their recovery goals and support them to implement these steps within the RMSS. For example, the RMSS can recommend an embedded urge surfing activity when someone’s immediate risk is driven by strong craving whereas a guided relaxation video can be provided to the user when they report stressful events. Similarly, the RMSS can encourage (and explicitly support) the user to reflect on recent past successes and/or skills they have developed when their self-efficacy is low.

The 2-week lagged model provides individuals with advanced warning of their lapse risk. This model is well-suited to support recovery needs that cannot be addressed immediately within an RMSS app, such as scheduling positive or pleasant activities, increasing social engagement, or attending a peer-led recovery meeting. To be clear, we do not believe an RMSS app alone will be sufficient to deliver continuing care. We expect individuals will require additional support throughout their recovery from a mental health provider (e.g., motivational enhancement, crisis management, skill building), a peer (e.g., sponsor, support group), or family member. Importantly, these types of supports take time to set up, highlighting the value of this lagged 2-week model.

At this point, it is still unclear the best way to provide risk and support information from our models to people. For an RMSS to be successful, users must trust the system, consistently engage with the system over time, and find the system beneficial. We have recently launched an NIAAA funded project to optimize daily support messages by examining the impact of several key message components (e.g., lapse probability, locally important features, a risk-relevant recovery activity recommendation, the linguistic style and tone of the message) on engagement, trust, clinical outcomes [67].

For a system using lagged models, we can imagine that lags longer than two weeks (i.e., more advanced warning) would be better still. In the present study, we could not train models with lags longer than two weeks because participants only provided lapse reports for up to three months. With the 2-week lagged model, we had approximately 17% fewer labeled observations for training because the last two weeks (out of 12 weeks) of labels for each participant were discarded. This data loss may be one factor that contributed to the decreases in model performance with increases in lag time and we believed that greater data loss (e.g., 25% for a 3-week lag) would not be tenable. We have recently completed data collection on a NIDA funded project where participants provided EMA and other sensed data for up to 12 months [20]. These data will allow us to train models with longer lags and to better evaluate the impact of data loss on model performance because lag time can be increased substantially with proportionally less data loss given 52 weeks of labeled observations per participant.

While the number of individual observations (i.e., # prediction timepoints per participant X # participants) was sufficient to train models with low bias, low variance and overall high performance, the relatively small number of unique participants remains an important limitation of this study. Our target sample size was calculated using traditional power analysis methods for logistic regression [30] because comparable approaches for machine learning models have not yet been validated. As a result, this estimate may not be as precise for our modeling context. Moreover, successful machine learning models must generalize well to new data (i.e., new observations among new individuals). Our analyses only speak to how well our models generalize to a small number of new individuals and these individuals are mostly homogeneous with respect to key demographic characteristics. To be clear, we made the most efficient use of our sample (i.e., using nested cross-validation to maximize the amount of held-out/test sets available for model evaluation); however, concerns about the generalizability to new individuals and other populations (e.g., individuals living in rural locations, members of other diverse groups not adequately represented in our sample) remain.

Our models predicted goal-inconsistent alcohol use. In our sample, in which all participants had a goal of abstinence, goal-inconsistent use was a homogeneous, observable behavior (i.e., any alcohol use). However, not all individuals in recovery choose complete abstinence. It remains an open question whether our predictors of goal-inconsistent use would generalize to alternative recovery goals (e.g., moderation). Future studies could allow individuals to self-define goal-inconsistent use (e.g., drinking on a weekday or drinking more than planned) and train models in samples with diverse recovery goals.

Similarly, many individuals in recovery from AUD also use other substances, and some may meet criteria for an additional substance use disorder. Because we did not measure the use of substances other than alcohol on our EMA, we were unable to include these behaviors as predictors of alcohol lapse in our models. Future research should consider how other substance use behaviors (and the extent to which they align with individuals’ recovery goals) impact alcohol lapse risk.

Our EMA measured 10 risk-relevant constructs. However, the relapse prevention literature also places strong emphasis on the role of protective constructs and their interaction with risk factors. Thus, our models might benefit from measuring protective constructs such as coping behaviors, social support, and other forms of recovery capital. Our EMA items also used a bipolar continuous measure of negative and positive affect. Although negative and positive affect are highly correlated, research suggests that they are distinct constructs with a nuanced relationship to alcohol use and lapse [68,69]. Measuring these constructs separately may improve lapse prediction while also providing a better understanding of the role of affect in alcohol lapse.

Our use of features from 4x daily EMA as model inputs may also raise concerns about measurement burden. We confirmed that participants can comply with such EMA schedules over this time period and that they find it acceptable given its potential benefits to them [19,see also 70]. However, frequent daily surveys may become too burdensome within an RMSS intended for use over many months to years for long-term continuing care. We have begun to address this concern by training no lag models with fewer EMAs (1x daily) and have found comparable performance [48]. Additionally, reinforcement learning could potentially be used for adaptive EMA sampling. For example, each day the algorithm could make a decision to send out an EMA or not based on inferred latent states of the individual based on previous EMA responses and predicted probability of lapse.

We have also begun to explore how we can supplement our models with data from lower burden sensing methods. Geolocation, which can be passively sensed, could compliment EMA well [71]. First, it could provide insight into information not easily captured by self-report without lengthy surveys. For example, the amount of time spent in risky locations, or changes in routine (e.g., loss of job; move to new city) that could indicate life stressors can be detected in movement patterns. Second, the near-continuous sampling of geolocation could offer risk-relevant information that would otherwise be missed in between the discrete sampling periods of EMA. Furthermore, potentially powerful features can be engineered by combining geolocation data with contextual information available in public sources (e.g., census data, alcohol outlet density) [72,73] or collected from the user directly (e.g., self-evaluated riskiness of a given location) [20].

Lastly, there may be value in exploring alternative modeling approaches that better leverage the temporal structure and individual-level nature of these data. For example, hierarchical models that start with population-based information (i.e., priors) and are updated with person-specific information as the individual provides data may be the most promising approach for effective use of time-varying information to model how an individual’s lapse risk evolves over time. Our lab has begun to develop these models and found preliminary evidence to suggest that they may outperform traditional population-level machine learning models [48]. However, in another recent study, population-level models well outperformed person-specific models for predicting various outcomes, including alcohol use and binge drinking [74]. Thus, the benefit of individualized models above and beyond population models remains an empirical question and is something we plan to continue to pursue in future work.

## Conclusions

This study suggests it is possible to accurately predict alcohol lapses both immediately and up to two weeks into the future using lagged machine learning prediction models. The no lag model could guide users to engage with a smart RMSS that provides daily recovery activities that are personalized to their lapse risk and the factors contributing to that risk. The 2-week lagged model could enable patients to seek out and implement recovery support that is not immediately available to them within the RMSS. Several important steps remain prior to implementing the no lag and 2-week lagged models within a smart RMSS. Feedback and support messages from these models should be optimized to sustain system engagement, trust, and clinical outcomes. Passive sensing of model inputs may allow assessment of a broader range of risk factors with less burden for system users. Perhaps most important, model fairness must be improved by decreasing disparities in performance for less privileged groups. We remain optimistic about the potential to implement these models within a smart RMSS because these barriers, while challenging, are surmountable.

## Supporting information

S1 Appendix Supporting tables and figures. (HTML)
