# Supplementary material for: Forecasting alcohol lapse risk up to two weeks in advance using time-lagged machine learning models
Source: PLoS One. 2026 Sep 8;21(9):e0356893. doi: 10.1371/journal.pone.0356893 (PMC13552771; doi:10.1371/journal.pone.0356893)
Supplement: Supplementary file 1 [file pone.0356893.s003.pdf]

Below, we delineate the reviewer's and editor's questions, concerns, and requests regarding our original submission, and we detail the ways in which we have addressed each item. All comments have been copied verbatim to avoid misinterpretation.

Editor

1. Please ensure that your manuscript meets PLOS ONE's style requirements, including those for file naming.

We have reviewed the PLOS One's style requirements and updated our manuscript accordingly. We have also ensured that our file names meet the requirements.

2. Please note that PLOS One has specific guidelines on code sharing for submissions in which author-generated code underpins the findings in the manuscript. In these cases, we expect all author-generated code to be made available without restrictions upon publication of the work.

We have uploaded a copy of our code to our OSF page following the recommended guidelines for code sharing. This OSF page is also referenced in our manuscript.

3. Thank you for stating the following financial disclosure: "This research was supported by grants from the National Institute on Alcohol Abuse and Alcoholism: https://www.niaaa.nih.gov/ (R01 AA024391 to JJC) and the National Institute on Drug Abuse: https://nida.nih.gov/ (R01 DA047315 to JJC)." Please state what role the funders took in the study. If the funders had no role, please state: "The funders had no role in study design, data collection and analysis, decision to publish, or preparation of the manuscript." If this statement is not correct you must amend it as needed. Please include this amended Role of Funder statement in your cover letter; we will change the online submission form on your behalf.

Our updated financial disclosure is documented below and also in the cover letter:

```
"This research was supported by grants from the National Institute on Alcohol Abuse and Alcoholis
```

4. PLOS requires an ORCID iD for the corresponding author in Editorial Manager on papers submitted after December 6th, 2016. Please ensure that you have an ORCID iD and that it is validated in Editorial Manager. To do this, go to 'Update my Information' (in the upper left-hand corner of the main menu), and click on the Fetch/Validate link next to the ORCID field. This will take you to the ORCID site and allow you to create a new iD or authenticate a pre-existing iD in Editorial Manager.

We have confirmed the ORCID ID is validated for the corresponding author.

5. Please include captions for your Supporting Information files at the end of your manuscript, and update any in-text citations to match accordingly.

We have included captions for our supporting information files at the end of the manuscript as requested.

6. If the reviewer comments include a recommendation to cite specific previously published works, please review and evaluate these publications to determine whether they are relevant and should be cited. There is no requirement to cite these works unless the editor has indicated otherwise.

N/A

Reviewer 1

1. The introduction mentions the capability of machine learning models to provide insights into individualized risk factors, leading to tailored momentary supports. Does the present model in the study enable this? Is it possible to generate feature importance rankings for given individuals?

Yes, we can generate local feature importance rankings (Shapley values) for every observation from every participant in our dataset. We previously reported global feature importance values, which average across all local Shapley values to provide a general global description of how important a feature is, on average, across all observations from all participants.

We have now, however, added a second method to better summarize the local feature importance values. Specifically, we calculate the proportion of observations in which each feature category has the highest Shapley value. This approach describes how frequently a feature category is the most influential contributor to individual predictions.

2. Related, a justification for not conducting model training within-persons is needed. To deploy these models for interventions, it seems that a "base" model trained on many people is important, but ideally this model can adapt when it is applied to a given individual (i.e., learning from incorrect predictions during the first few weeks).

When outcomes (such as lapse events) are rare, person-specific models cannot be built until the individual has experienced several positive outcome labels (i.e., lapses). It may take weeks, months, or longer to accrue enough positive labels to calculate auROC in a nested cross-validation framework. We agree with this reviewer that a population model that can learn from new data from a specific individual may be a promising alternative approach. However, whether this approach yields better predictive performance still remains an empirical question. We have begun exploring how priors from a population model can be gradually downweighted as individual-level data are collected and become more important, and our preliminary findings suggest that this approach may outperform population-level models. However, other studies have found that person-specific models may substantially underperform compared to population-level models. We now discuss this approach and its potential implications in the Discussion section.

3. Could you provide sensitivity and specificity of the final model? These performance metrics are common in machine learning and easier to interpret than auROC (especially for non-ML audiences). Perhaps prediction labels could be made for discrete 24-hour windows, leading to day-level sensitivity/specificity. Accuracy may also be helpful.

We did not previously report sensitivity, specificity, and accuracy because these are properties of class predictions. They are metrics calculated at a specific decision threshold. A decision threshold is only necessary when a "decision" about whether to intervene must be made. We intend for our model's predicted probabilities to be used to provide risk-relevant information to an individual irregardless of

their predicted risk level. Thus, we believe that assigning a class label of lapse versus no lapse would be less clinically useful (compared to these full-range probability scores) and may even be potentially iatrogenic (e.g., triggering an abstinence violation effect). Moreover, the optimal decision threshold is likely to vary substantially depending on the clinical context and the relative costs of misclassification. For these reasons, we use auROC, which characterizes the "accuracy" of probabilities as the primary focus in the paper. Nonetheless, we now also report sensitivity, specificity, and balanced accuracy at a single decision threshold (identified with Youden's index) in a supplemental table. We chose to use balanced accuracy instead of accuracy because accuracy can be misleading in highly imbalanced datasets. Balanced accuracy corrects for imbalanced data by calculating accuracy for each class separately (i.e., sensitivity and specificity) and then averaging them.

4. Does model sensitivity change as an individual drinks more (i.e., sensitivity to detect first lapse versus second, and so forth)? This may have implications regarding within-person training and the reliability of the model overtime as an individual proceeds through recovery.

We have now added an additional analysis and supplemental figure to explore differences in model performance for predicting an individual's first lapse compared to later lapses. Specifically, we calculated auROC separately for first and later lapses across the 30 held-out test sets. Individuals who never lapsed while on study were included in both groups to keep sample sizes relatively equivalent.

We found that, overall, the model performed worse for first lapses than for later lapses, with performance remaining relatively stable across lag times (median auROCs of 0.78–0.79). In contrast, the model performed very well in predicting later lapses (median auROCs of 0.87–0.94). Performance for later lapses showed a moderate decline as lag time increased, mirroring the pattern observed in overall model performance.

This suggests that our models do extremely well at predicting lapses that occur later sequentially. This is not surprising given that past alcohol use predicts future alcohol use quite well. Additionally, these findings may indicate that the model is capturing meaningful within-person changes across the recovery trajectory.

Regarding the lower performance on predicting first lapses, this may not be problematic. Lapses are common in recovery and a single lapse does not constitute relapse. However, following a lapse is a vulnerable period where an individual might continue to lapse (and possibly return to previous levels of hazardous drinking) or initiate supports to re-engage with their recovery goals. Clinically, lapses are often referred to as "teachable moments" where opportunities to intervene are well-received. Thus, even if we underestimate risk for the first lapse, the following period may still provide a timely window for accurate risk monitoring and recovery support.

5. Please provide a definition of "feature scoring epoch" when the term is first introduced. (Line 225)

We have updated our language so that feature scoring epoch is now only used to describe the windows in which features are calculated (i.e., 12, 24, 48, 72, and 168 hours). This is described in the Feature Engineering section. In the Predictions section (including line 225) and the accompanying Figure 1 we have removed this terminology.

6. The cross-validation approach has many facets and jargon. A narrative description of this process may be more digestible, and potentially an accompanying figure.

We now open with a narrative description of nested cross-validation in te Cross-validation section. We also have simplified our text and avoided using technical jargon when possible.

7. How was 'past alcohol use' measured? More detail is needed on how this was measured, particularly regarding timing and amount, especially given its feature importance.

Past use was measured on the first item of the EMA. This item prompted participants to enter the start and end dates and times of any unreported past alcohol use. We now more clearly describe the measurement of this item in the Measures section.

We have also added a paragraph to the Feature Engineering section that explicitly details how past use features were engineered from this EMA item.

"We calculated raw and difference rate features for past use from reported lapses on the EMA. Raw past use features were generated by dividing the total number of previously reported lapses within a feature scoring epoch by the duration of that epoch. For difference past use rate features, we subtracted the baseline past use rate for that participant (i.e., total number of lapses while on study divided by total hours on study before the prediction window) from their associated raw past use rate. We also calculated raw and difference rate features for completed EMAs (i.e., number of full EMA surveys that were completed in a feature scoring epoch divided by the duration of that epoch)."

8. In the abstract, the description around the timing of the prediction is confusing. "predicting next day lapses up to two weeks in advance". Does this mean prediction of the next day or something that happens in two weeks? Another example is line 105-106: "predicting lapse probability in a 24-hour window that begins two weeks in the future). It is not clear what begins two weeks in the future?

Our models provided predicted probabilities of an alcohol lapse in a future 24-hour period (the prediction window). For the 2-week lagged model, that future prediction window starts 2 weeks from the prediction timepoint and ends two weeks and 1 day from the prediction timepoint. We have updated the abstract, introduction (lines 105-106), and methods (see Predictions section) to make this more clear.

9. A definition for early recovery at first mention would be helpful.

We now provide a definition for early recovery (<= 8 weeks of abstinence) at first mention in the Participants section and in the abstract.

10. In the discussion, a consideration for how the results may or may not transfer to a sample as those with harm reduction (versus abstinence) goals would improve the implications

We appreciate this suggestion and have added a paragraph to the Discussion section that considers how lapse, as defined for individuals choosing abstinence, differs from lapse for individuals pursuing other recovery goals, as well as the implications for the generalizability of our models.

11. How was compensation prorated for compliance?

Compensation was not prorated based on compliance. Participants were compensated $25 for each month they provided EMA data with less than 10% missingness. We now make this compensation scheme more clear in the methods and provide details on other forms of compensation during the study (e.g., payments to offset cellular plans).

12. Was other substance use considered as a predictor?

We did not collect information about other substance use on our EMA and therefore could not include this as a predictor in our models.

13. There is some question regarding the clinical utility of the 2 – week lag, could that be discussed?

We hope we have made the clinical utility of the 2-week lagged model more clear with our revisions to related to point 8. Given that our 2-week model predicts the probability of a lapse that occurs within a 24-hour prediction window that starts two weeks from the prediction timepoint, this model gives patients 2 weeks to implement changes or engage in recovery activities to address their risk. The focus of previous alcohol lapse prediction research has been on predicting immediate lapses (i.e., the probability of a lapse in a prediction window that starts at the prediction timepoint). This can be helpful, for engaging in an activity in an app, but many recovery supports (e.g., attending a self-help group, scheduling a therapy session, or making plans to meet up with a supportive friend) are not available immediately. If patients knew their risk of lapse was going to be high on a given day two-weeks from now they could schedule the activities that would be most helpful to them in advance.
