# Supplementary material for: Forecasting alcohol lapse risk up to two weeks in advance using time-lagged machine learning models
Source: PLoS One. 2026 Sep 8;21(9):e0356893. doi: 10.1371/journal.pone.0356893 (PMC13552771; doi:10.1371/journal.pone.0356893)
Supplement: Supplementary file 2 [file pone.0356893.s004.pdf]

Below, we delineate the reviewer's and editor's questions, concerns, and requests regarding our original submission, and we detail the ways in which we have addressed each item.

Editor

1. The abstract is three paragraphs long. Please condense it into a single paragraph. Please also add keywords.

We condensed the abstract into a single paragraph and added keywords.

2. Line 155: missing full stop.

We added a full stop to the end of line 155.

3. Please justify why participants exhibiting severe psychosis and paranoia were excluded, but not those experiencing severe symptoms of other mental health conditions.

We now elaborate on this study protocol decision. This psychosis/paranoia exclusion criterion was implemented because the study involved the collection of sensitive data without real-time clinical monitoring or treatment, and individuals experiencing severe psychotic symptoms may require a higher level of clinical support than the study protocol was designed to provide. We did not exclude participants based on the presence of severe symptoms of other mental health disorders because we aimed to recruit a sample representative of the broader clinical population, in which AUD commonly co-occurs with other psychiatric disorders. Excluding participants on the basis of other psychiatric symptoms would have substantially reduced the representativeness of the sample and limited the generalizability of the findings.

4. Please move the Ethics statement above the Procedure section.

We moved the ethics statement above the procedure section.

5. Please include as a limitation that you did not collect information on other substance use during the EMA and therefore could not consider it as a predictor.

We now add this a limitation and discuss its implications in the discussion section.

6. Please include as a limitation that the sample size calculations may not be precise, as approaches for machine learning methodologies have not yet been validated.

We now add this as a limitation to the discussion section.

7. Line 357: missing closing bracket.

We added the missing closet bracket to line 357.

Reviewer 1

1. I noticed just one small typo on line 32 where "assessment" was cut, I think was likely just intended to be plural?

We added "assessments" where it was previously missing in line 32.
