# Supplementary material for: Forecasting alcohol lapse risk up to two weeks in advance using time-lagged machine learning models
Source: PLoS One. 2026 Sep 8;21(9):e0356893. doi: 10.1371/journal.pone.0356893 (PMC13552771; doi:10.1371/journal.pone.0356893)
Supplement: Supplementary file 3 [file pone.0356893.s005.pdf]

Below, we delineate the concerns and requests regarding our original submission, and we detail the ways in which we have addressed each item.

**1. Please acknowledge in the limitations that positive and negative affect were assessed using a continuous scale. There is evidence to suggest that positive and negative affect are separate, though highly correlated, constructs; therefore, using continuous scale may overlook some of the nuanced ways in which affect is associated with other constructs.**

We now address this as a limitation in our discussion. See paragraph starting on line 744.

**2. On p. 35, line 724, you refer to @hsiehSampleSizeTables1989. I assume this is a reference that was not properly converted. Please correct this.**

We have corrected this reference formatting error.
