# Supplementary material for: Forecasting alcohol lapse risk up to two weeks in advance using time-lagged machine learning models
Source: PLoS One. 2026 Sep 8;21(9):e0356893. doi: 10.1371/journal.pone.0356893 (PMC13552771; doi:10.1371/journal.pone.0356893)
Supplement: Supplementary file 4 [file pone.0356893.s001.html]

Supplemental Material


# Supplemental Material

Author

Kendra Wyant, Gaylen E. Fronk, Jiachen Yu, Claire E. Punturieri, and John J. Curtin

Published

May 22, 2026

This file contains the supplemental materials for *Forecasting Risk of Alcohol Lapse up to Two Weeks in Advance using Time-lagged Machine Learning Models*. It includes all supplemental figures and tables. Additional materials are made available on our study’s OSF page (https://osf.io/xta67/).

---

## 1 Supplemental Figures

### 1.1 Figure A. Model performance by model and timing of lapse

To understand differences in model performance for predicting an individual’s first lapse compared to later lapses, we calculated median area under the receiver operating characteristic curve (auROC) with Bayesian priors and the 95% Bayesian credible intervals separately for first and later lapses across the 30 held-out test sets. Individuals who never lapsed while on study were included in both groups to keep sample sizes relatively equivalent.

Compared to overall model performance (.85-.91), models performed less well when predicting an individual’s first lapse (median auROCs ranging from .78-.79; Figure A). This performance was relatively consistent across lag times. Conversely, models performed better when predicting later lapses (.87-.94), compared to overall performance. Performance for later lapses showed a moderate decline as lag time increased, mirroring the pattern seen in overall model performance (Figure 1 in main manuscript).

Posterior probabilities for area under the receiver operating curve (auROC) by model (No lag, 1-day, 3-day, 1-week, 2-week) and timing of lapse (first vs. later lapse). auROC ranges from .5 (chance performance) to 1 (perfect performance). Median posterior probabilities over 12,000 posterior samples (4 chains × 3,000 samples) are depicted as points. Vertical lines represent the 95% Bayesian credible intervals for the posterior distribution.

### 1.2 Figure B. Calibration plots for all models

Figure B shows the calibration plots for the raw and calibrated probabilities for all five models. It also includes a histogram of raw probabilities along the x-axis to illustrate range of predictions and bin size.

Calibration plots of raw and calibrated predicted lapse probabilities (beta, isotonic, and logistic) across models (no lag, 1-day, 3-day, 1-week, and 2-week lag). Predicted probabilities (x-axis) are binned into deciles. Observed lapse probability (y-axis) represents the proportion of actual lapses observed in each bin. The dashed diagonal represents perfect calibration. Points below the line indicate overestimation and points above the line indicate underestimation. Raw probabilities are depicted as the solid black curves. Platt calibrated probabilites are depicted as the pink dashed curves. The grey histogram along the bottom of the plot represents the proportion of raw probabilities in each bin.

### 1.3 Figure C. Global Shapley values as a function of lag time

Global feature importance is an indicator of how important a feature category was to the model’s predictions, on average (i.e., across all participants and all observations). In Figure C, we present the global feature importance values across lag times. The magnitude of importance for our two most important features, past use and future efficacy, sharply declined as lag time increased. There were also moderate increases in feature importance for risk situations and valence/arousal with increased lag time.

Global importance (mean |Shapley value|) for feature categories by lag time.

### 1.4 Figure D. Global Shapley plots for baseline and 2-week lag models by demographic subgroup

Figure D presents the global feature importance values separately by demographic subgroup for the no lag and 2-week lag models. The relative ordering of important features remained consistent across groups.

Figure 1: Global importance (mean |Shapley value|) for feature categories for no lag and 2-week lagged model by demographic group. The importance of each feature category for advantaged and disadvantaged demographic groups are displayed separately by color.

## 2 Supplemental Tables

### 2.1 Table A. Classification performance metrics

In Table A we report median sensitivity, specificity, and balanced accuracy calculated at a single decision threshold (identified using Youden’s index) across the 30 held-out test sets separately for each model. We calculated these metrics using the Platt-scaled probabilities to provide insight into how well the models can correctly label a prediction window as lapse or no lapse. However, we caution against using classification with these models. A decision threshold is only necessary when a “decision” about whether to intervene must be made. We intend for our model’s predicted probabilities to be used to provide risk-relevant information to an individual irregardless of their predicted risk level. We believe that assigning a class label of lapse versus no lapse would be less clinically useful (compared to these full-range probability scores) and may even be potentially iatrogenic. Moreover, the optimal decision threshold is likely to vary substantially depending on the clinical context and the relative costs of misclassification.

Table 1: Sensitivity, specificity, and balanced accuracy performance estimates for each model at a specific decision threshold identified by Youden’s index.

| model | sensitivity | specificity | balanced accuracy |
| --- | --- | --- | --- |
| No lag | 0.84 | 0.83 | 0.84 |
| 1-day | 0.84 | 0.80 | 0.82 |
| 3-day | 0.85 | 0.80 | 0.82 |
| 1-week | 0.86 | 0.75 | 0.81 |
| 2-week | 0.85 | 0.73 | 0.78 |

### 2.2 Table B. Demographic Contrasts for all Models

Table B presents the median difference in auROC, 95% Bayesian CI, and posterior probability that the auROC difference was smaller than 0 for the three fairness contrasts: race/ethnicity (non-White and/or Hispanic; *N* = 20 vs. Non-Hispanic White; *N* = 131), sex at birth (female; *N* = 74 vs. male; *N* = 77), and income (below poverty line; *N* = 49 vs. above poverty line; *N* = 102). Median auROC differences less than 0 indicate the model, on average, performed worse for the non-advantaged group (female, non-White and/or Hispanic, below poverty line) compared to the advantaged group (male, non-Hispanic White, and above poverty line) for all models.

Table 2: This table reports the bayesian model contrasts for each demographic group comparison separately by model and contrast. The median value represents the difference in median area under the ROC curve (auROC) between the two groups. A negative value indicates lower auROC performance for the disadvantaged group (female, non-White and/or Hispanic, income below poverty line) compared to the advantaged group (male, non-Hispanic White, income above poverty line).

|  | Sex at birth | | | Income | | | Race/Ethnicity | | |
| --- | --- | --- | --- | --- | --- | --- | --- | --- | --- |
| Lag | Median | Bayesian CI | Probability | Median | Bayesian CI | Probability | Median | Bayesian CI | Probability |
| No lag | -0.043 | [-0.059, -0.028] | 1 | -0.012 | [-0.033, 0.007] | 0.848 | -0.131 | [-0.222, -0.057] | 0.999 |
| 1-day | -0.044 | [-0.063, -0.027] | 1 | -0.02 | [-0.04, -0.001] | 0.955 | -0.165 | [-0.28, -0.072] | 0.999 |
| 3-day | -0.051 | [-0.072, -0.031] | 1 | -0.023 | [-0.045, -0.002] | 0.961 | -0.157 | [-0.236, -0.087] | 1 |
| 1-week | -0.08 | [-0.103, -0.059] | 1 | -0.012 | [-0.037, 0.012] | 0.807 | -0.125 | [-0.204, -0.057] | 0.999 |
| 2-week | -0.098 | [-0.125, -0.073] | 1 | -0.039 | [-0.073, -0.008] | 0.98 | -0.13 | [-0.208, -0.058] | 0.998 |

### 2.3 Table C. Brier scores for all models

The raw probabilities produced by our final models were not well calibrated. Consequently, we used Platt scaling to improve calibration. Table C presents Brier scores for the raw and Platt scaled probabilities for each model.

Brier scores for raw and Platt-scaled probabilities by classification model (No lag, 1-day, 3-day, 1-week, 2-week).

| Model | Raw (uncalibrated) | Platt calibration |
| --- | --- | --- |
| No lag | 0.07 | 0.04 |
| 1-day | 0.07 | 0.05 |
| 3-day | 0.13 | 0.06 |
| 1-week | 0.07 | 0.06 |
| 2-week | 0.08 | 0.06 |
