# Supplementary material for: Forecasting alcohol lapse risk up to two weeks in advance using time-lagged machine learning models
Source: PLoS One. 2026 Sep 8;21(9):e0356893. doi: 10.1371/journal.pone.0356893 (PMC13552771; doi:10.1371/journal.pone.0356893)
Supplement: Peer review history

# Peer review: Decision Letter 0

**DOI:** 10.1371/journal.pone.0356893.r001

**Authors:** Ilse Bloom

28 Apr 2026

Dear Dr. Curtin,

Thank you for submitting your manuscript to PLOS ONE. After careful consideration, we feel that it has merit but does not fully meet PLOS ONE’s publication criteria as it currently stands. Therefore, we invite you to submit a revised version of the manuscript that addresses the points raised during the review process.

Please submit your revised manuscript by Jun 11 2026 11:59PM. If you will need more time than this to complete your revisions, please reply to this message or contact the journal office at plosone@plos.org. When you're ready to submit your revision, log on to https://www.editorialmanager.com/pone/ and select the 'Submissions Needing Revision' folder to locate your manuscript file.

- A letter that responds to each point raised by the academic editor and reviewer(s). You should upload this letter as a separate file labeled 'Response to Reviewers'.
- A marked-up copy of your manuscript that highlights changes made to the original version. You should upload this as a separate file labeled 'Revised Manuscript with Track Changes'.
- An unmarked version of your revised paper without tracked changes. You should upload this as a separate file labeled 'Manuscript'.

If you would like to make changes to your financial disclosure, please include your updated statement in your cover letter. Guidelines for resubmitting your figure files are available below the reviewer comments at the end of this letter.

If applicable, we recommend that you deposit your laboratory protocols in protocols.io to enhance the reproducibility of your results. Protocols.io assigns your protocol its own identifier (DOI) so that it can be cited independently in the future. For instructions see: https://journals.plos.org/plosone/s/submission-guidelines#loc-laboratory-protocols. Additionally, PLOS ONE offers an option for publishing peer-reviewed Lab Protocol articles, which describe protocols hosted on protocols.io. Read more information on sharing protocols at https://plos.org/protocols?utm_medium=editorial-email&utm_source=authorletters&utm_campaign=protocols.

As the corresponding author, your ORCID iD is verified in the submission system and will appear in the published article. PLOS supports the use of ORCID, and we encourage all coauthors to register for an ORCID iD and use it as well. Please encourage your coauthors to verify their ORCID iD within the submission system before final acceptance, as unverified ORCID iDs will not appear in the published article. *Only*  the individual author can complete the verification step; PLOS staff *cannot*  verify ORCID iDs on behalf of authors.

We look forward to receiving your revised manuscript.

Kind regards,

Ilse Bloom

Staff Editor

PLOS One

Journal Requirements:

When submitting your revision, we need you to address these additional requirements.

1.Please ensure that your manuscript meets PLOS ONE's style requirements, including those for file naming. The PLOS ONE style templates can be found at

https://journals.plos.org/plosone/s/file?id=wjVg/PLOSOne_formatting_sample_main_body.pdf and

https://journals.plos.org/plosone/s/file?id=ba62/PLOSOne_formatting_sample_title_authors_affiliations.pdf

2. Please note that PLOS One has specific guidelines on code sharing for submissions in which author-generated code underpins the findings in the manuscript. In these cases, we expect all author-generated code to be made available without restrictions upon publication of the work. Please review our guidelines at https://journals.plos.org/plosone/s/materials-and-software-sharing#loc-sharing-code and ensure that your code is shared in a way that follows best practice and facilitates reproducibility and reuse.

3. Thank you for stating the following financial disclosure:

“This research was supported by grants from the National Institute on Alcohol Abuse and Alcoholism: https://www.niaaa.nih.gov/ (R01 AA024391 to JJC) and the National Institute on Drug Abuse: https://nida.nih.gov/ (R01 DA047315 to JJC).”

Please state what role the funders took in the study.  If the funders had no role, please state: "The funders had no role in study design, data collection and analysis, decision to publish, or preparation of the manuscript."

If this statement is not correct you must amend it as needed.

Please include this amended Role of Funder statement in your cover letter; we will change the online submission form on your behalf.

4. PLOS requires an ORCID iD for the corresponding author in Editorial Manager on papers submitted after December 6th, 2016. Please ensure that you have an ORCID iD and that it is validated in Editorial Manager. To do this, go to ‘Update my Information’ (in the upper left-hand corner of the main menu), and click on the Fetch/Validate link next to the ORCID field. This will take you to the ORCID site and allow you to create a new iD or authenticate a pre-existing iD in Editorial Manager.

5. Please include captions for your Supporting Information files at the end of your manuscript, and update any in-text citations to match accordingly. Please see our Supporting Information guidelines for more information: http://journals.plos.org/plosone/s/supporting-information.

6.If the reviewer comments include a recommendation to cite specific previously published works, please review and evaluate these publications to determine whether they are relevant and should be cited. There is no requirement to cite these works unless the editor has indicated otherwise.

Additional Editor Comments:

Please note that we have only been able to secure a single reviewer to assess your manuscript. We are issuing a decision on your manuscript at this point to prevent further delays in the evaluation of your manuscript. Please be aware that the editor who handles your revised manuscript might find it necessary to invite additional reviewers to assess this work once the revised manuscript is submitted. However, we will aim to proceed on the basis of this single review if possible.

The comments from the reviewer can be found below. Please review these comments and make the appropriate revisions to your manuscript alongside a point-by-point response to the concerns raised.

[Note: HTML markup is below. Please do not edit.]

Reviewers' comments:

Reviewer's Responses to Questions

**Comments to the Author**

1. Is the manuscript technically sound, and do the data support the conclusions?

Reviewer #1: Yes

**********

2. Has the statistical analysis been performed appropriately and rigorously?

Reviewer #1: Yes

**********

3. Have the authors made all data underlying the findings in their manuscript fully available?

The PLOS Data policy

Reviewer #1: Yes

**********

4. Is the manuscript presented in an intelligible fashion and written in standard English?

Reviewer #1: Yes

**********

Reviewer #1: Thank you for the opportunity to review this interesting manuscript, which used machine learning to predict alcohol lapses in a sample of 151 individuals in early recovery. Results suggested a high auROC for all models and showed a few important group differences. The models did not perform as well for non-advantaged groups and there were also a few interesting features that predicted drinking (previous alcohol use, self-efficacy, and craving). This work has important implications for using model prediction to improve recovery monitoring and in future interventions. However, there are a few important considerations before the manuscript will be suitable for publication.

1. The introduction mentions the capability of machine learning models to provide insights into individualized risk factors, leading to tailored momentary supports. Does the present model in the study enable this? Is it possible to generate feature importance rankings for given individuals?

2. Related, a justification for not conducting model training within-persons is needed. To deploy these models for interventions, it seems that a “base” model trained on many people is important, but ideally this model can adapt when it is applied to a given individual (i.e., learning from incorrect predictions during the first few weeks).

3. Could you provide sensitivity and specificity of the final model? These performance metrics are common in machine learning and easier to interpret than auROC (especially for non-ML audiences). Perhaps prediction labels could be made for discrete 24-hour windows, leading to day-level sensitivity/specificity. Accuracy may also be helpful.

4. Does model sensitivity change as an individual drinks more (i.e., sensitivity to detect first lapse versus second, and so forth)? This may have implications regarding within-person training and the reliability of the model overtime as an individual proceeds through recovery.

5. Please provide a definition of “feature scoring epoch” when the term is first introduced. (Line 225)

6. The cross-validation approach has many facets and jargon. A narrative description of this process may be more digestible, and potentially an accompanying figure.

7. How was ‘past alcohol use’ measured? More detail is needed on how this was measured, particularly regarding timing and amount, especially given its feature importance.

8. In the abstract, the description around the timing of the prediction is confusing. “predicting next day lapses up to two weeks in advance”. Does this mean prediction of the next day or something that happens in two weeks? Another example is line 105-106: “predicting lapse probability in a 24-hour window that begins two weeks in the future). It is not clear what begins two weeks in the future?

9. A definition for early recovery at first mention would be helpful.

10. In the discussion, a consideration for how the results may or may not transfer to a sample as those with harm reduction (versus abstinence) goals would improve the implications

11. How was compensation prorated for compliance?

12. Was other substance use considered as a predictor?

13. There is some question regarding the clinical utility of the 2 – week lag, could that be discussed?

**********

what does this mean?). If published, this will include your full peer review and any attached files.

If you choose “no”, your identity will remain anonymous but your review may still be made public.

**Do you want your identity to be public for this peer review?**  For information about this choice, including consent withdrawal, please see our Privacy Policy

Reviewer #1: No

**********

[NOTE: If reviewer comments were submitted as an attachment file, they will be attached to this email and accessible via the submission site. Please log into your account, locate the manuscript record, and check for the action link "View Attachments". If this link does not appear, there are no attachment files.]

To ensure your figures meet our technical requirements, please review our figure guidelines: https://journals.plos.org/plosone/s/figures

You may also use PLOS’s free figure tool, NAAS, to help you prepare publication quality figures: https://journals.plos.org/plosone/s/figures#loc-tools-for-figure-preparation.

NAAS will assess whether your figures meet our technical requirements by comparing each figure against our figure specifications.

# Author comment: Author response to Decision Letter 1

**DOI:** 10.1371/journal.pone.0356893.r002

8 Jun 2026

Below, we delineate the reviewer's and editor’s questions, concerns, and requests regarding our original submission, and we detail the ways in which we have addressed each item. All comments have been copied verbatim to avoid misinterpretation.

Editor

1. Please ensure that your manuscript meets PLOS ONE's style requirements, including those for file naming.

We have reviewed the PLOS One's style requirements and updated our manuscript accordingly. We have also ensured that our file names meet the requirements.

<!--https://journals.plos.org/plosone/s/file?id=wjVg/PLOSOne_formatting_sample_main_body.pdf

https://journals.plos.org/plosone/s/file?id=ba62/PLOSOne_formatting_sample_title_authors_affiliations.pdf

2. Please note that PLOS One has specific guidelines on code sharing for submissions in which author-generated code underpins the findings in the manuscript. In these cases, we expect all author-generated code to be made available without restrictions upon publication of the work.

We have uploaded a copy of our code to our OSF page following the recommended guidelines for code sharing. This OSF page is also referenced in our manuscript.

3. Thank you for stating the following financial disclosure: “This research was supported by grants from the National Institute on Alcohol Abuse and Alcoholism: https://www.niaaa.nih.gov/ (R01 AA024391 to JJC) and the National Institute on Drug Abuse: https://nida.nih.gov/ (R01 DA047315 to JJC).” Please state what role the funders took in the study. If the funders had no role, please state: "The funders had no role in study design, data collection and analysis, decision to publish, or preparation of the manuscript." If this statement is not correct you must amend it as needed. Please include this amended Role of Funder statement in your cover letter; we will change the online submission form on your behalf.

Our updated financial disclosure is documented below and also in the cover letter:

“This research was supported by grants from the National Institute on Alcohol Abuse and Alcoholism: https://www.niaaa.nih.gov/ (R01 AA024391 to JJC) and the National Institute on Drug Abuse: https://nida.nih.gov/ (R01 DA047315 to JJC). The funders had no role in study design, data collection and analysis, decision to publish, or preparation of the manuscript."

4. PLOS requires an ORCID iD for the corresponding author in Editorial Manager on papers submitted after December 6th, 2016. Please ensure that you have an ORCID iD and that it is validated in Editorial Manager. To do this, go to ‘Update my Information’ (in the upper left-hand corner of the main menu), and click on the Fetch/Validate link next to the ORCID field. This will take you to the ORCID site and allow you to create a new iD or authenticate a pre-existing iD in Editorial Manager.

We have confirmed the ORCID ID is validated for the corresponding author.

5. Please include captions for your Supporting Information files at the end of your manuscript, and update any in-text citations to match accordingly.

We have included captions for our supporting information files at the end of the manuscript as requested.

6. If the reviewer comments include a recommendation to cite specific previously published works, please review and evaluate these publications to determine whether they are relevant and should be cited. There is no requirement to cite these works unless the editor has indicated otherwise.

N/A

Reviewer 1

1. The introduction mentions the capability of machine learning models to provide insights into individualized risk factors, leading to tailored momentary supports. Does the present model in the study enable this? Is it possible to generate feature importance rankings for given individuals?

Yes, we can generate local feature importance rankings (Shapley values) for every observation from every participant in our dataset. We previously reported global feature importance values, which average across all local Shapley values to provide a general global description of how important a feature is, on average, across all observations from all participants.

We have now, however, added a second method to better summarize the local feature importance values. Specifically, we calculate the proportion of observations in which each feature category has the highest Shapley value. This approach describes how frequently a feature category is the most influential contributor to individual predictions.

2. Related, a justification for not conducting model training within-persons is needed. To deploy these models for interventions, it seems that a “base” model trained on many people is important, but ideally this model can adapt when it is applied to a given individual (i.e., learning from incorrect predictions during the first few weeks).

When outcomes (such as lapse events) are rare, person-specific models cannot be trained until the individual has experienced several positive outcome labels (i.e., lapses). It may take weeks, months, or longer to accrue enough positive labels to calculate auROC in a nested cross-validation framework. We agree with this reviewer that a population model that can learn from new data from a specific individual may be a promising alternative approach. However, whether this approach yields better predictive performance still remains an empirical question. We have begun exploring how priors from a population model can be gradually downweighted as individual-level data are collected and become more important, and our preliminary findings suggest that this approach may outperform population-level models. However, other studies have found that person-specific models may substantially under-perform compared to population-level models. We now discuss this approach and its potential implications in the Discussion section.

3. Could you provide sensitivity and specificity of the final model? These performance metrics are common in machine learning and easier to interpret than auROC (especially for non-ML audiences). Perhaps prediction labels could be made for discrete 24-hour windows, leading to day-level sensitivity/specificity. Accuracy may also be helpful.

We did not previously report sensitivity, specificity, and accuracy because these are properties of class predictions. They are metrics calculated at a specific decision threshold. A decision threshold is only necessary when a "decision" about whether to intervene must be made. We intend for our model's predicted probabilities to be used to provide risk-relevant information to an individual irregardless of their predicted risk level. Thus, we believe that assigning a class label of lapse versus no lapse would be less clinically useful (compared to these full-range probability scores) and may even be potentially iatrogenic (e.g., triggering an abstinence violation effect). Moreover, the optimal decision threshold is likely to vary substantially depending on the clinical context and the relative costs of misclassification. For these reasons, we use auROC, which characterizes the "accuracy" of probabilities as the primary focus in the paper. Nonetheless, we now also report sensitivity, specificity, and balanced accuracy at a single decision threshold (identified with Youden's index) in a supplemental table. We chose to use balanced accuracy instead of accuracy because accuracy can be misleading in highly imbalanced datasets. Balanced accuracy corrects for imbalanced data by calculating accuracy for each class separately (i.e., sensitivity and specificity) and then averaging them.

4. Does model sensitivity change as an individual drinks more (i.e., sensitivity to detect first lapse versus second, and so forth)? This may have implications regarding within-person training and the reliability of the model overtime as an individual proceeds through recovery.

We have now added an additional analysis and supplemental figure to explore differences in model performance for predicting an individual's first lapse compared to later lapses. Specifically, we calculated auROC separately for first and later lapses across the 30 held-out test sets. Individuals who never lapsed while on study were included in both groups to keep sample sizes relatively equivalent.

We found that, overall, the model performed worse for first lapses than for later lapses, with performance remaining relatively stable across lag times (median auROCs of 0.78–0.79). In contrast, the model performed very well in predicting later lapses (median auROCs of 0.87–0.94). Performance for later lapses showed a moderate decline as lag time increased, mirroring the pattern observed in overall model performance.

This suggests that our models do extremely well at predicting lapses that occur later sequentially. This is not surprising given that past alcohol use predicts future alcohol use quite well. Additionally, these findings may indicate that the model is capturing meaningful within-person changes across the recovery trajectory.

Regarding the lower performance on predicting first lapses, this may not be problematic. Lapses are common in recovery and a single lapse does not constitute relapse. However, following a lapse is a vulnerable period where an individual might continue to lapse (and possibly return to previous levels of hazardous drinking) or initiate supports to re-engage with their recovery goals. Clinically, lapses are often referred to as "teachable moments" where opportunities to intervene are well-received. Thus, even if we underestimate risk for the first lapse, the following period may still provide a timely window for accurate risk monitoring and recovery support.

5. Please provide a definition of “feature scoring epoch” when the term is first introduced. (Line 225)

We have updated our language so that feature scoring epoch is now only used to describe the windows in which features are calculated (i.e., 12, 24, 48, 72, and 168 hours). This is described in the Feature Engineering section. In the Predictions section (including line 225) and the accompanying Figure 1 we have removed this terminology.

6. The cross-validation approach has many facets and jargon. A narrative description of this process may be more digestible, and potentially an accompanying figure.

We now open with a narrative description of nested cross-validation in te Cross-validation section. We also have simplified our text and avoided using technical jargon when possible.

7. How was ‘past alcohol use’ measured? More detail is needed on how this was measured, particularly regarding timing and amount, especially given its feature importance.

Past use was measured on the first item of the EMA. This item prompted participants to enter the start and end dates and times of any unreported past alcohol use. We now more clearly describe the measurement of this item in the Measures section.

We have also added a paragraph to the Feature Engineering section that explicitly details how past use features were engineered from this EMA item.

"We calculated raw and difference rate features for past use from reported lapses on the EMA. Raw past use features were generated by dividing the total number of previously reported lapses within a feature scoring epoch by the duration of that epoch. For difference past use rate features, we subtracted the baseline past use rate for that participant (i.e., total number of lapses while on study divided by total hours on study before the prediction window) from their associated raw past use rate. We also calculated raw and difference rate features for completed EMAs (i.e., number of full EMA surveys that were completed in a feature scoring epoch divided by the duration of that epoch)."

8. In the abstract, the description around the timing of the prediction is confusing. “predicting next day lapses up to two weeks in advance”. Does this mean prediction of the next day or something that happens in two weeks? Another example is line 105-106: “predicting lapse probability in a 24-hour window that begins two weeks in the future). It is not clear what begins two weeks in the future?

Our models provided predicted probabilities of an alcohol lapse in a future 24-hour period (the prediction window). For the 2-week lagged model, that future prediction window starts 2 weeks from the prediction timepoint and ends two weeks and 1 day from the prediction timepoint. We have updated the abstract, introduction (lines 105-106), and methods (see Predictions section) to make this more clear.

9. A definition for early recovery at first mention would be helpful.

We now provide a definition for early recovery (<= 8 weeks of abstinence) at first mention in the Participants section and in the abstract.

10. In the discussion, a consideration for how the results may or may not transfer to a sample as those with harm reduction (versus abstinence) goals would improve the implications

We appreciate this suggestion and have added a paragraph to the Discussion section that considers how lapse, as defined for individuals choosing abstinence, differs from lapse for individuals pursuing other recovery goals, as well as the implications for the generalizability of our models.

11. How was compensation prorated for compliance?

Compensation was not prorated based on compliance. Participants were compensated \$25 for each month they provided EMA data with less than 10% missingness. We now make this compensation scheme more clear in the methods and provide details on other forms of compensation during the study (e.g., payments to offset cellular plans).

12. Was other substance use considered as a predictor?

We did not collect information about other substance use on our EMA and therefore could not include this as a predictor in our models.

13. There is some question regarding the clinical utility of the 2 – week lag, could that be discussed?

We hope we have made the clinical utility of the 2-week lagged model more clear with our revisions to related to point 8. Given that our 2-week model predicts the probability of a lapse that occurs within a 24-hour prediction window that starts two weeks from the prediction timepoint, this model gives patients 2 weeks to implement changes or engage in recovery activities to address their risk. The focus of previous alcohol lapse prediction research has been on predicting immediate lapses (i.e., the probability of a lapse in a prediction window that starts at the prediction timepoint). This can be helpful, for engaging in an activity in an app, but many recovery supports (e.g., attending a self-help group, scheduling a therapy session, or making plans to meet up with a supportive friend) are not available immediately. If patients knew their risk of lapse was going to be high on a given day two-weeks from now they could schedule the activities that would be most helpful to them in advance.

Attachment Submitted filename: reviewer_responses.pdf

# Peer review: Decision Letter 1

**DOI:** 10.1371/journal.pone.0356893.r003

**Authors:** Anna Tovmasyan

10 Jul 2026

Dear Dr. Curtin,

Thank you for submitting your manuscript to PLOS ONE. After careful consideration, we feel that it has merit but does not fully meet PLOS ONE’s publication criteria as it currently stands. Therefore, we invite you to submit a revised version of the manuscript that addresses the points raised during the review process.

Please submit your revised manuscript by Aug 24 2026 11:59PM. If you will need more time than this to complete your revisions, please reply to this message or contact the journal office at plosone@plos.org. When you're ready to submit your revision, log on to https://www.editorialmanager.com/pone/ and select the 'Submissions Needing Revision' folder to locate your manuscript file.

- A letter that responds to each point raised by the academic editor and reviewer(s). You should upload this letter as a separate file labeled 'Response to Reviewers'.
- A marked-up copy of your manuscript that highlights changes made to the original version. You should upload this as a separate file labeled 'Revised Manuscript with Track Changes'.
- An unmarked version of your revised paper without tracked changes. You should upload this as a separate file labeled 'Manuscript'.

If you would like to make changes to your financial disclosure, please include your updated statement in your cover letter. Guidelines for resubmitting your figure files are available below the reviewer comments at the end of this letter.

If applicable, we recommend that you deposit your laboratory protocols in protocols.io to enhance the reproducibility of your results. Protocols.io assigns your protocol its own identifier (DOI) so that it can be cited independently in the future. For instructions see: https://journals.plos.org/plosone/s/submission-guidelines#loc-laboratory-protocols. Additionally, PLOS ONE offers an option for publishing peer-reviewed Lab Protocol articles, which describe protocols hosted on protocols.io. Read more information on sharing protocols at https://plos.org/protocols?utm_medium=editorial-email&utm_source=authorletters&utm_campaign=protocols.

As the corresponding author, your ORCID iD is verified in the submission system and will appear in the published article. PLOS supports the use of ORCID, and we encourage all coauthors to register for an ORCID iD and use it as well. Please encourage your coauthors to verify their ORCID iD within the submission system before final acceptance, as unverified ORCID iDs will not appear in the published article. *Only*  the individual author can complete the verification step; PLOS staff *cannot*  verify ORCID iDs on behalf of authors.

We look forward to receiving your revised manuscript.

Kind regards,

Anna Tovmasyan

Academic Editor

PLOS One

Journal Requirements:

If the reviewer comments include a recommendation to cite specific previously published works, please review and evaluate these publications to determine whether they are relevant and should be cited. There is no requirement to cite these works unless the editor has indicated otherwise.

Please review your reference list to ensure that it is complete and correct. If you have cited papers that have been retracted, please include the rationale for doing so in the manuscript text, or remove these references and replace them with relevant current references. Any changes to the reference list should be mentioned in the rebuttal letter that accompanies your revised manuscript. If you need to cite a retracted article, indicate the article’s retracted status in the References list and also include a citation and full reference for the retraction notice.

Additional Editor Comments:

Please address the following:

- The abstract is three paragraphs long. Please condense it into a single paragraph. Please also add keywords.

- Line 155: missing full stop.

- Please justify why participants exhibiting severe psychosis and paranoia were excluded, but not those experiencing severe symptoms of other mental health conditions.

- Please move the Ethics statement above the Procedure section.

- Please include as a limitation that you did not collect information on other substance use during the EMA and therefore could not consider it as a predictor.

- Please include as a limitation that the sample size calculations may not be precise, as approaches for machine learning methodologies have not yet been validated.

- Line 357: missing closing bracket.

[Note: HTML markup is below. Please do not edit.]

Reviewers' comments:

Reviewer's Responses to Questions

**Comments to the Author**

Reviewer #1: All comments have been addressed

**********

2. Is the manuscript technically sound, and do the data support the conclusions?

Reviewer #1: Yes

**********

3. Has the statistical analysis been performed appropriately and rigorously?

Reviewer #1: Yes

**********

4. Have the authors made all data underlying the findings in their manuscript fully available?

The PLOS Data policy

Reviewer #1: Yes

**********

5. Is the manuscript presented in an intelligible fashion and written in standard English?

Reviewer #1: Yes

**********

Reviewer #1: The authors have adequately addressed all prior comments. I noticed just one small typo on line 32 where "assessment" was cut, I think was likely just intended to be plural?

**********

what does this mean?). If published, this will include your full peer review and any attached files.

If you choose “no”, your identity will remain anonymous but your review may still be made public.

**Do you want your identity to be public for this peer review?**  For information about this choice, including consent withdrawal, please see our Privacy Policy

Reviewer #1: No

**********

[NOTE: If reviewer comments were submitted as an attachment file, they will be attached to this email and accessible via the submission site. Please log into your account, locate the manuscript record, and check for the action link "View Attachments". If this link does not appear, there are no attachment files.]

To ensure your figures meet our technical requirements, please review our figure guidelines: https://journals.plos.org/plosone/s/figures

You may also use PLOS’s free figure tool, NAAS, to help you prepare publication quality figures: https://journals.plos.org/plosone/s/figures#loc-tools-for-figure-preparation.

NAAS will assess whether your figures meet our technical requirements by comparing each figure against our figure specifications.

# Author comment: Author response to Decision Letter 2

**DOI:** 10.1371/journal.pone.0356893.r004

18 Jul 2026

Editor

1. The abstract is three paragraphs long. Please condense it into a single paragraph. Please also

add keywords.

We condensed the abstract into a single paragraph and added keywords.

2. Line 155: missing full stop.

We added a full stop to the end of line 155.

3. Please justify why participants exhibiting severe psychosis and paranoia were excluded, but not

those experiencing severe symptoms of other mental health conditions.

We now elaborate on this study protocol decision. This psychosis/paranoia exclusion criterion was

implemented because the study involved the collection of sensitive data without real-time clinical moni-

toring or treatment, and individuals experiencing severe psychotic symptoms may require a higher level

of clinical support than the study protocol was designed to provide. We did not exclude participants

based on the presence of severe symptoms of other mental health disorders because we aimed to recruit

a sample representative of the broader clinical population, in which AUD commonly co-occurs with

other psychiatric disorders. Excluding participants on the basis of other psychiatric symptoms would

have substantially reduced the representativeness of the sample and limited the generalizability of the

findings.

4. Please move the Ethics statement above the Procedure section.

We moved the ethics statement above the procedure section.

5. Please include as a limitation that you did not collect information on other substance use

during the EMA and therefore could not consider it as a predictor.

We now add this a limitation and discuss its implications in the discussion section.

6. Please include as a limitation that the sample size calculations may not be precise, as

approaches for machine learning methodologies have not yet been validated.

We now add this as a limitation to the discussion section.

7. Line 357: missing closing bracket.

We added the missing closet bracket to line 357.

Reviewer 1

1. I noticed just one small typo on line 32 where “assessment” was cut, I think was likely just

intended to be plural?

We added “assessments” where it was previously missing in line 32.

Attachment Submitted filename: reviewer_responses_auresp_2.pdf

# Editor report: Decision Letter 2

**DOI:** 10.1371/journal.pone.0356893.r005

**Authors:** Anna Tovmasyan

22 Jul 2026

Dear Dr. Curtin,

Thank you for submitting your manuscript to PLOS ONE. After careful consideration, we feel that it has merit but does not fully meet PLOS ONE’s publication criteria as it currently stands. Therefore, we invite you to submit a revised version of the manuscript that addresses the points raised during the review process.

Thank you for addressing the concerns raised. There are only two minor issues that need to be resolved prior to publication.

First, please acknowledge in the limitations that positive and negative affect were assessed using a continuous scale. There is evidence to suggest that positive and negative affect are separate, though highly correlated, constructs; therefore, using continuous scale may overlook some of the nuanced ways in which affect is associated with other constructs.

Second, on p. 35, line 724, you refer to @hsiehSampleSizeTables1989. I assume this is a reference that was not properly converted. Please correct this.

Please submit your revised manuscript by Sep 05 2026 11:59PM. If you will need more time than this to complete your revisions, please reply to this message or contact the journal office at plosone@plos.org. When you're ready to submit your revision, log on to https://www.editorialmanager.com/pone/ and select the 'Submissions Needing Revision' folder to locate your manuscript file.

- A letter that responds to each point raised by the academic editor and reviewer(s). You should upload this letter as a separate file labeled 'Response to Reviewers'.
- A marked-up copy of your manuscript that highlights changes made to the original version. You should upload this as a separate file labeled 'Revised Manuscript with Track Changes'.
- An unmarked version of your revised paper without tracked changes. You should upload this as a separate file labeled 'Manuscript'.

If you would like to make changes to your financial disclosure, please include your updated statement in your cover letter. Guidelines for resubmitting your figure files are available below the reviewer comments at the end of this letter.

If applicable, we recommend that you deposit your laboratory protocols in protocols.io to enhance the reproducibility of your results. Protocols.io assigns your protocol its own identifier (DOI) so that it can be cited independently in the future. For instructions see: https://journals.plos.org/plosone/s/submission-guidelines#loc-laboratory-protocols. Additionally, PLOS ONE offers an option for publishing peer-reviewed Lab Protocol articles, which describe protocols hosted on protocols.io. Read more information on sharing protocols at https://plos.org/protocols?utm_medium=editorial-email&utm_source=authorletters&utm_campaign=protocols.

As the corresponding author, your ORCID iD is verified in the submission system and will appear in the published article. PLOS supports the use of ORCID, and we encourage all coauthors to register for an ORCID iD and use it as well. Please encourage your coauthors to verify their ORCID iD within the submission system before final acceptance, as unverified ORCID iDs will not appear in the published article. *Only*  the individual author can complete the verification step; PLOS staff *cannot*  verify ORCID iDs on behalf of authors.

We look forward to receiving your revised manuscript.

Kind regards,

Anna Tovmasyan

Academic Editor

PLOS One

Journal Requirements:

If the reviewer comments include a recommendation to cite specific previously published works, please review and evaluate these publications to determine whether they are relevant and should be cited. There is no requirement to cite these works unless the editor has indicated otherwise.

Please review your reference list to ensure that it is complete and correct. If you have cited papers that have been retracted, please include the rationale for doing so in the manuscript text, or remove these references and replace them with relevant current references. Any changes to the reference list should be mentioned in the rebuttal letter that accompanies your revised manuscript. If you need to cite a retracted article, indicate the article’s retracted status in the References list and also include a citation and full reference for the retraction notice.

[Note: HTML markup is below. Please do not edit.]

To ensure your figures meet our technical requirements, please review our figure guidelines: https://journals.plos.org/plosone/s/figures

You may also use PLOS’s free figure tool, NAAS, to help you prepare publication quality figures: https://journals.plos.org/plosone/s/figures#loc-tools-for-figure-preparation.

NAAS will assess whether your figures meet our technical requirements by comparing each figure against our figure specifications.

# Author comment: Author response to Decision Letter 3

**DOI:** 10.1371/journal.pone.0356893.r006

6 Aug 2026

1. Please acknowledge in the limitations that positive and negative affect were assessed using a continuous scale. There is evidence to suggest that positive and negative affect are separate, though highly correlated, constructs; therefore, using continuous scale may overlook some of the nuanced ways in which affect is associated with other constructs.

We now address this as a limitation in our discussion. See paragraph starting on line 744.

2. On p. 35, line 724, you refer to @hsiehSampleSizeTables1989. I assume this is a reference that was not properly converted. Please correct this.

We have corrected this reference formatting error.

Attachment Submitted filename: reviewer_responses_auresp_3.pdf

# Editor report: Decision Letter 3

**DOI:** 10.1371/journal.pone.0356893.r007

**Authors:** Anna Tovmasyan

10 Aug 2026

Forecasting alcohol lapse risk up to two weeks in advance using time-lagged machine learning models

PONE-D-25-65933R3

Dear Dr. Curtin,

We’re pleased to inform you that your manuscript has been judged scientifically suitable for publication and will be formally accepted for publication once it meets all outstanding technical requirements.

Within one week, you’ll receive an e-mail detailing the required amendments. When these have been addressed, you’ll receive a formal acceptance letter and your manuscript will be scheduled for publication.

An invoice will be generated when your article is formally accepted. Please note, if your institution has a publishing partnership with PLOS and your article meets the relevant criteria, all or part of your publication costs will be covered. Please make sure your user information is up-to-date by logging into Editorial Manager at Editorial Manager® and clicking the ‘Update My Information' link at the top of the page. For questions related to billing, please contact billing support.

If your institution or institutions have a press office, please notify them about your upcoming paper to help maximize its impact. If they’ll be preparing press materials, please inform our press team as soon as possible -- no later than 48 hours after receiving the formal acceptance. Your manuscript will remain under strict press embargo until 2 pm Eastern Time on the date of publication. For more information, please contact onepress@plos.org.

Kind regards,

Anna Tovmasyan

Academic Editor

PLOS One

# Editor report: Acceptance letter

**DOI:** 10.1371/journal.pone.0356893.r008

**Authors:** Anna Tovmasyan

PONE-D-25-65933R3

PLOS One

Dear Dr. Curtin,

I'm pleased to inform you that your manuscript has been deemed suitable for publication in PLOS One. Congratulations! Your manuscript is now being handed over to our production team.

At this stage, our production department will prepare your paper for publication. This includes ensuring the following:

* All references, tables, and figures are properly cited

* All relevant supporting information is included in the manuscript submission,

* There are no issues that prevent the paper from being properly typeset

You will receive further instructions from the production team, including instructions on how to review your proof when it is ready. Please keep in mind that we are working through a large volume of accepted articles, so please give us a few days to review your paper and let you know the next and final steps.

Lastly, if your institution or institutions have a press office, please let them know about your upcoming paper now to help maximize its impact. If they'll be preparing press materials, please inform our press team within the next 48 hours. Your manuscript will remain under strict press embargo until 2 pm Eastern Time on the date of publication. For more information, please contact onepress@plos.org.

You will receive an invoice from PLOS for your publication fee after your manuscript has reached the completed accept phase. If you receive an email requesting payment before acceptance or for any other service, this may be a phishing scheme. Learn how to identify phishing emails and protect your accounts at https://explore.plos.org/phishing.

If we can help with anything else, please email us at customercare@plos.org.

Thank you for submitting your work to PLOS One and supporting open access.

Kind regards,

PLOS ONE Editorial Office Staff

on behalf of

Dr. Anna Tovmasyan

Academic Editor

PLOS One
